# Revealing the hidden diversity of *Gyrodactylus* communities (Monogenea, Gyrodactylidae) from Nearctic Catostomidae and Leuciscidae fish hosts (Teleostei, Cypriniformes), with descriptions of ten new species[Fn FN1]

**DOI:** 10.1051/parasite/2023035

**Published:** 2023-09-27

**Authors:** Chahrazed Rahmouni, Mária Seifertová, Andrea Šimková

**Affiliations:** Department of Botany and Zoology, Faculty of Science, Masaryk University Kotlářská 2 611 37 Brno Czech Republic

**Keywords:** Monogenea, *Gyrodactylus*, North America, Species diversity, Haptor morphology, DNA diversity

## Abstract

Despite the high diversity of freshwater fishes in the Nearctic region, little is known about the composition of their parasite communities. We addressed the diversity of viviparous monogeneans of *Gyrodactylus* parasitizing highly diversified cypriniform fish inhabiting Nearctic watersheds. Nowadays, a thorough assessment of *Gyrodactylus* spp. diversity requires both morphological traits and genetic data. A combination of taxonomically important haptoral features and sequences of the ITS regions and 18S rDNA revealed 25 *Gyrodactylus* spp. parasitizing two catostomid and 15 leuciscid species sampled in six distinct localities in the United States and Canada. These include ten *Gyrodactylus* species recognized as new to science and described herein (*G. ellae* n. sp., *G. hamdii* n. sp., *G. hanseni* n. sp., *G. huyseae* n. sp., *G. kuchtai* n. sp., *G. lummei* n. sp., *G. mendeli* n. sp., *G. prikrylovae* n. sp., *G. scholzi* n. sp., and *G. steineri* n. sp.), seven already known species, and finally eight undescribed species. Overall, Nearctic *Gyrodactylus* spp. exhibited haptoral morphotypes known from fish hosts worldwide and those apparently restricted to Nearctic *Gyrodactylus* lineages like the typical ventral bar with a median knob and a plate-like membrane, or the additional filament attached to the handles of marginal hooks. The integrative approach further evidenced possible ongoing gene flow, host-switching in generalist *Gyrodactylus* spp., and regional translocation of monogenean fauna through fish introductions. The study highlights the hitherto underexplored morphological and genetic diversity of viviparous monogeneans throughout the Nearctic region.

## Abstract (fr)

Malgré la grande diversité des poissons d’eau douce dans la région néarctique, on sait peu de choses sur la composition de leurs communautés de parasites. Nous avons abordé la diversité des monogènes vivipares du genre *Gyrodactylus* parasitant des poissons cypriniformes très diversifiés habitant les bassins versants néarctiques. De nos jours, une évaluation approfondie de la diversité de *Gyrodactylus* spp. nécessite à la fois des traits morphologiques et des données génétiques. Une combinaison de caractéristiques haptorales et de séquences taxonomiquement importantes des régions ITS et de l’ADNr 18S a révélé 25 espèces de *Gyrodactylus* sur deux espèces de catostomidés et 15 espèces de leuciscidés échantillonnées dans six localités distinctes aux États-Unis et au Canada. Celles-ci comprennent dix espèces de *Gyrodactylus* reconnues comme nouvelles pour la science et décrites dans cette étude (*G. ellae* n. sp., *G. hamdii* n. sp., *G. hanseni* n. sp., *G. huyseae* n. sp., *G. kuchtai* n. sp., *G. lummei* n. sp., *G. mendeli* n. sp., *G. prikrylovae* n. sp., *G. scholzi* n. sp. et *G. steineri* n. sp.), sept espèces déjà connues et enfin huit espèces non décrites. Dans l’ensemble, les espèces de *Gyrodactylus* néarctiques présentaient des morphotypes haptoraux connus chez les poissons hôtes du monde entier et ceux apparemment limités aux lignées néarctiques de *Gyrodactylus* comme la barre ventrale typique avec un bouton médian et une membrane en forme de plaque, ou le filament supplémentaire attaché aux poignées des crochets marginaux. L’approche intégrative a en outre mis en évidence le flux de gènes en cours, le changement d’hôte chez les espèces de *Gyrodactylus* généralistes et la translocation régionale de la faune monogène par l’introduction de poissons. L’étude met en évidence la diversité morphologique et génétique jusqu’ici sous-explorée des monogènes vivipares dans toute la région néarctique.

## Introduction

The North American continent hosts one of the most diverse temperate freshwater fish faunas in the world, with several thousand described and numerous undescribed species [28, 56]. With a wide geographical distribution, cypriniforms compose the most diverse monophyletic freshwater fish clades [63, 73], counting over 4000 species [28]. Cyprinoidei, the most speciose lineage of cypriniforms, comprises Cyprinidae (carps and minnows) and Leuciscidae (true minnows) as the largest and most diverse groups [92]. Overall, over 80 genera were established for leuciscids [28], distributed in Nearctic and Palearctic Eurasia [6, 64]. Leuciscidae is a single cyprinoid family naturally distributed in North America. Cypriniformes in the Holarctic region are also represented by suckers (Catostomidae, Catostomoidei), with 13 catostomid genera native to North America and a single genus native to Asia [38, 100, 101].

Monogeneans are highly host-specific parasites [105], reflecting the distribution of their hosts across continents [51, 94]. *Gyrodactylus* von Nordmann, 1832 (Gyrodactylidae Cobbold, 1864) is a well-known, highly diverse monogenean genus with almost 500 known species parasitizing teleost fish [1, 8], including some highly pathogenic species [2]. While life history traits of most *Gyrodactylus* flatworms predominantly remain unknown, *Gyrodactylus* spp. have been recognized to parasitize representatives of almost 20 bony fish orders and exhibit a variable degree of host specificity [3, 51, 80, 105]. This might be linked to the direct life cycle and the lack of a specialized transmission stage, which favors host switching, in contrast to limited host choices that face the larval stage (oncomiracidia) of oviparous monogeneans [45]. Members of *Gyrodactylus* are known for their site specificity: they are present on external surfaces like skin and fins (for instance *G. atratuli* Putz & Hoffman, 1963 [84]), restricted to the gills only (for instance *G. baeacanthus* Wellborn & Rogers, 1967 [103]), or present on the skin, fins and gills as well (for instance *G. corleonis* Paladini, Cable Fioravanti, Faria & Shinn, 2010 [78]).

In general, the description of any monogenean species based on morphological characters alone can be problematic and requires considerable expertise. Morphologically, *Gyrodactylus* spp. show inconspicuous diversity with relatively little variations in their attachment apparatus, termed the haptor. Although Malmberg [59] elaborated a morphological method of *Gyrodactylus* classification based on the excretory system, the discrimination of gyrodactylid taxa remains problematic. Malmberg’s “species-group” concept was for a long time regarded as the miracle approach for separating species, but this view was later challenged when genetic data recovered the *G. wageneri* group as paraphyletic [9], while morphology and host preference suggested monophyly [3]. In addition, sclerotized haptoral features in *Gyrodactylus* (anchors, transverse bars and marginal hooks) may vary ecophenotypically depending on parasite age, season, geographic distribution, location on host, and host species (see, for instance, [25, 26]).

The integration of methods other than genetics for discriminating *Gyrodactylus* spp. has not always been successful. This was the case, for instance, with the application of statistical classifiers on high-quality scanning electron micrographs obtained from *G. salaris* Malmberg, 1957 and *G. thymalli* Zitnan, 1960, two well-known pathogenic species from salmonids [93]. On the contrary, the combination of traditional morphological characterizations and DNA sequences has been shown to be efficient to a certain degree in *Gyrodactylus* spp. delineation (see, for instance, [43, 61]). However, in the case of *G. salaris* and *G. thymalli*, almost no genetic variation was observed using the internal transcribed spacer (ITS; ITS1-5.8S-ITS2) regions of rDNA [16, 109], whereas, later, these two species were shown to be conspecific with microRNA loci analyses [30]. The ITS fragments evidenced variations between *G. salaris*, *G. derjavini* Mikailov, 1975, and *G. truttae* Gläser, 1974 parasitizing salmonids [16]. A few other genetic markers, such as the ribosomal intergenic spacer (IGS) and cytochrome *c* oxidase subunit I (COI), were shown to be useful in terms of revealing genetic variation compared to ITS sequences [17, 34–36, 67].

*Gyrodactylus* spp. have a worldwide distribution in freshwater, brackish, and marine habitats [4], and mostly parasitize cypriniform fishes [1, 37]. *Gyrodactylus*, with more than 50 currently known species, represents the second largest monogenean genus known from Nearctic fishes. Leuciscids in Palearctic and Nearctic regions harbor different species of *Gyrodactylus* (except for a few co-introduced species in North America) [51].

In recent decades, research targeting the parasite fauna in Nearctic freshwater fishes has lagged behind similar research in Europe [91]. While North America possesses a higher diversity of cypriniform fishes than Europe, the known parasite species richness, specifically that of monogeneans and tapeworms per cypriniform species in Europe, is much higher compared to North America [51].

In light of the lack of knowledge on current fish parasite diversity [51], our study was specifically focused on viviparous monogeneans of Nearctic cypriniform fish fauna with the aim of recovering the hidden diversity of *Gyrodactylus* communities in broadly diversified Leuciscidae and Catostomidae. We applied an integrative approach to combine morphological characters and molecular markers.

## Material and methods

### Fish host collection and identification

Cypriniform fish hosts were collected in 2018, 2019, and 2022 from distinct freshwater systems in the United States (Arkansas, New York, Mississippi, and Wisconsin) and Canada (Quebec). Information related to cypriniform fish hosts, their sampling localities, and *Gyrodactylus* diversity is shown in Table 1. Fish identification was performed by local collaborators (listed in acknowledgements) or based on common identification keys. Fieldwork was carried out with the approval of the official local authorities (provided to US partners).

**Table 1:** List of cypriniform species investigated in the present study, grouped by host suborders, sample size, total body length, and localities of sampling in the Nearctic region, and list of *Gyrodactylus* species identified in fish hosts. USA: United States of America; CA: Canada.

Cypriniform suborder	Family	Species	*n* host	River basin	Water body	State/Province	Total length TL (cm)	*Gyrodactylus* spp.
Catostomoidei	Catostomidae	*Catostomus catostomus* Rafinesque, 1820	1	Atlantic Ocean	Cap-Rouge River	Quebec (CA)	9.5_1_	*G. wardi*
		*Catostomus commersonii* (Lacepède, 1803)	5	Mid Atlantic	Rom Hill Beaver Pond	Cooperstown, New York (USA)	18.8 ± 5 (14–24)_4_	*G. ellae* n. sp.
			2	Mid Atlantic	Leatherstocking Creek	Otsego, New York (USA)	11.1 ± 0.3 (10.9–11.3)_2_	*G. spathulatus*
			3	Atlantic Ocean	Cap-Rouge River	Quebec (CA)	7.8 ± 1.4 (7–9.5)_3_	*G. hamdii* n. sp.
Cyprinoidei	Leuciscidae	*Campostoma spadiceum* (Girard, 1856)	1	Arkansas-White-Red	Butcherknife Creek	Polk County, Arkansas (USA)	–	*G. lummei* n. sp.
			4	Arkansas-White-Red	Big Fork Creek	Polk County, Arkansas (USA)	11.5 ± 1.4 (10–12.8)_4_	*G.* sp. 1 “*C. spadiceum*”
			3	Arkansas-White-Red	Rock Creek	Polk County, Arkansas (USA)	8.4 ± 0.9 (7.5–9.2)_3_	*G.* sp. 2 “*C. spadiceum*”
			3	Arkansas-White-Red	Caddo River	Polk County, Arkansas (USA)	8.8 ± 2.5 (7–10.5)_2_	
		*Chrosomus neogaeus* (Cope, 1867)	13	Great Lakes	Mink River	Door County, Wisconsin (USA)	6.2 ± 0.8 (4.7–7.8)_13_	*G. kuchtai* n. sp.
		*Clinostomus elongatus* (Kirtland, 1840)	1	Great Lakes	West Twin River	Brown County, Wisconsin (USA)	11.1_1_	*G. steineri* n. sp.
		*Cyprinella venusta* Girard, 1856	25	Pascagoula	Pascagoula River	Oxbow south of Cumbest bridge landing, Mississippi (USA)	6.6 ± 1.4 (4.5–10)_25_	*G.* sp. “*C. venusta*”
		*Exoglossum maxillingua* (Lesueur, 1817)	1	Mid Atlantic	Oaks Creek	Otsego, New York (USA)	11_1_	*G. colemanensis*
		*Hybognathus nuchalis* Agassiz, 1855	1	Pascagoula	Pascagoula River	Oxbow south of Cumbest bridge landing, Mississippi (USA)	6.5_1_	*G.* sp. “*H. nuchalis*”
		*Luxilus chrysocephalus* Rafinesque, 1820	2	Arkansas-White-Red	Ouachita Mountains Biological Station	Polk County, Arkansas (USA)	11_1_	*G. hanseni* n. sp.
			1	Arkansas-White-Red	Big Fork Creek	Polk County, Arkansas (USA)	–	*G. huyseae* n. sp.
			1	Arkansas-White-Red	Reed Creek	Polk County, Arkansas (USA)	11_1_	
			1	Arkansas-White-Red	Rock Creek	Polk County, Arkansas (USA)	11_1_	
		*Lythrurus* sp.	4	Arkansas-White-Red	Rock Creek	Polk County, Arkansas (USA)	6.6 ± 0.7 (5.8–7.3)_4_	*G.* sp. “*Lythrurus* sp.”
		*Notemigonus crysoleucas* (Mitchill, 1814)	1	Arkansas-White-Red	Rock Creek	Polk County, Arkansas (USA)	4.7_1_	*G. variabilis*
			5	Mid Atlantic	Rom Hill Beaver Pond	Cooperstown, New York (USA)	9.3 ± 0.7 (9–10.5)_5_	
			4	Atlantic Ocean	Saint- Augustine Lake	Quebec (CA)	13.2 ± 2.2 (10–15)_4_	
		*Nocomis biguttatus* (Kirtland, 1840)	5	Great Lakes	West Twin River	Brown County, Wisconsin (USA)	13.1 ± 2.8 (7.7–15.1)_5_	*G. mendeli* n. sp.
		*Notropis hudsonius* (Clinton, 1824)	4	Mid Atlantic	Rom Hill Beaver Pond	Cooperstown, New York (USA)	9.4 ± 0.9 (8.9–10.7)_4_	*G. huyseae* n. sp.
			2	Mid Atlantic	Chip Lake	Cooperstown, New York (USA)	7.5 ± 0.7 (7–8)_2_	
		*Pimephales promelas* Rafinesque, 1820	3	Mid Atlantic	Rom Hill Beaver Pond	Cooperstown, New York (USA)	6 ± 0.5 (5.6–6.5)_3_	*G. prikrylovae* n. sp.
			7	Great Lakes	Morrys Creek	Door County, Wisconsin (USA)	6.6 ± 0.8 (5.2–8)_7_	*G. scholzi* n. sp.
		*Rhinichthys atratulus* (Hermann, 1804)	1	Mid Atlantic	Oaks Creek	Otsego, New York (USA)	5.8_1_	*G.* sp. 1 “*R. atratulus*”
			2	Great Lakes	Mink River	Door County, Wisconsin (USA)	9.2 ± 0.07 (9.2 –9.3)_2_	*G.* sp. 2 “*R. atratulus*”
			1	Great Lakes	Morrys Creek	Door County, Wisconsin (USA)	9.3_1_	*G. atratuli*
			3	Mid Atlantic	Leatherstocking Creek	Otsego, New York (USA)	6.1 ± 0.3 (6–6.5)_3_	*G. stunkardi*
			1	Great Lakes	Morrys Creek	Door County, Wisconsin (USA)	8.6_1_	
		*Rhinichthys cataractae* (Valenciennes, 1842)	3	Mid Atlantic	Leatherstocking Creek	Otsego, New York (USA)	7.4 ± 1.4 (6.5–9)_3_	*G. atratuli*
			2	Atlantic Ocean	Cap Rouge River	Quebec (CA)	7.3 ± 2.5 (5.5–9)_2_	
			2	Mid Atlantic	Oaks Creek	Otsego, New York (USA)	7.8 ± 0.1 (7.7–7.8)_2_	*G. dechtiari*
		*Semotilus atromaculatus* (Mitchill, 1818)	1	Arkansas-White-Red	Ouachita Mountains Biological Station	Polk County, Arkansas (USA)	–	*G. hanseni* n. sp.
			1	Arkansas-White-Red	Butcherknife Creek,	Polk County, Arkansas (USA)	19.4_1_	
			1	Arkansas-White-Red	Big Fork Creek	Polk County, Arkansas (USA)	10.5_1_	
			1	Arkansas-White-Red	Caddo River	Polk County, Arkansas (USA)	–	
			2	Arkansas-White-Red	Bear Creek	Polk County, Arkansas (USA)	15 ± 3.4 (10.5–15.5)_2_	

The identity of the investigated cypriniform hosts was further checked by means of molecular barcoding using the partial cytochrome *b* (cyt-*b*) gene. Mitochondrial DNA of host species was isolated from fin clips preserved in 96% ethanol using a DNeasy^®^ Blood & Tissue Kit (QIAGEN, Hilden, Germany), following the manufacturer’s instructions. Amplification of the cyt-*b* gene was performed using forward primer GluF (5′–AACCACCGTTGTATTCAACTACAA–3′) and reverse primer ThrR (5′–ACCTCCGATCTTCGGATTACAAGACCG–3′) [57]. PCR reactions consisted of 1 U of Taq polymerase (Fermentas, Thermo Fisher Scientific, Waltham, MA, USA), 1 × PCR buffer, 1.5 mM MgCl_2_, 0.4 mM of each dNTP, 0.4 μM of each primer, and an aliquot of 30 ng (1 μL) of genomic DNA in a total volume of 25 μL. PCR was carried out in a Mastercycler ep gradient S (Eppendorf AG, Hamburg, Germany) with the following steps: 2 min at 94 °C followed by 39 cycles of 45 s at 92 °C, 90 s at 48 °C, and 105 s at 72 °C, and 7 min of final elongation at 72 °C. The PCR product was purified by ExoSAP-IT™ (Amplia, Bratislava, Slovakia) and was sequenced directly in both directions using the same primers as in the amplification reaction. The initial amplification was carried out using a BigDye^®^ Terminator v3.1 Cycle Sequencing Kit (Applied Biosystems by Thermo Fisher Scientific, Waltham, MA, USA) and an Applied Biosystems 3130 Genetic Analyzer (Applied Biosystems). Raw nucleotide sequences were edited using Sequencher software v. 5.0 (Gene Codes, Ann Arbor, MI, USA) and aligned using ClustalW [98] as implemented in MEGA v. 11 [97]. The identification of cypriniform species based on a sequence similarity approach was carried out using the Basic Local Alignment Search Tool (https://blast.ncbi.nlm.nih.gov/Blast.cgi: blastn, default settings). Newly generated sequences for the cypriniform species were deposited in GenBank (see the species descriptions below). Catostomid and leuciscid fish host nomenclature follows FishBase [29].

### Parasite collection and morphometric study

During the field trip, fins and gills were examined for *Gyrodactylus* spp. using an MST130 stereoscopic microscope. Monogenean specimens were removed using surgical needles and mounted on slides with a mixture of glycerine and ammonium picrate (GAP) [58]. Selected specimens of each collected monogenean species were cut in half using fine needles under a dissecting microscope. The anterior part of the body with male copulatory organ (MCO) was placed in a 1.5 mL Eppendorf tube with 96% ethanol for DNA extraction, while the posterior part with haptoral sclerites (anchors, bars and marginal hooks) was fixed in GAP for morphological characterization. *Gyrodactylus* spp. were identified using original descriptions (see the result sections for references). Measurements and photographs were taken using an Olympus BX51 phase-contrast microscope and Olympus Stream Image Analysis v. 1.9.3 software (Olympus, Tokyo, Japan). Measurements of *Gyrodactylus* spp. are shown in micrometers and are given as the mean followed by the range and the number of measurements (*n*) in parentheses. Drawings of the haptoral sclerotized parts were made on flattened specimens using an Olympus BX51 microscope equipped with a drawing tube and edited with a graphic tablet compatible with Adobe Illustrator CS6 v. 16.0.0 and Adobe Photoshop v. 13.0 (Adobe Systems Inc., San Jose, CA, USA). Infection indices were calculated for all collected *Gyrodactylus* spp. with a sufficient sample size (sample size for a few non-described species was very low, see below) according to [7]. The type-material was deposited in the National Museum of Natural History (MNHN, Paris, France) under accession numbers HEL1996–HEL2034.

### Genetic characterization

Each *Gyrodactylus* specimen preserved in 96% ethanol was dried using an Eppendorf 5301 Concentrator. Total genomic DNA was extracted using a DNeasy^®^ Blood & Tissue Kit following the protocol for the purification of total DNA from animal tissues. Two nuclear ribosomal DNA markers suitable for the differentiation of *Gyrodactylus* spp. were used (for instance, [9, 31, 61, 79, 111]). A fragment spanning ITS1, 5.8S and ITS2 (ITS regions) was amplified using forward primer ITS1F (5′-GTTTCCGTAGGTGAACCT -3′) [88], complementary to the sequence at the 3′ end of the 18S rRNA gene, and reverse primer ITS2 (5′-TCCTCCGCTTAGTGATA-3′), complementary to the sequence at the 5′ end of the 28S rRNA gene [16]. A partial fragment of 18S rDNA containing the V4 region, which exhibits intraspecific variation in *Gyrodactylus* [15, 61], was amplified using the primer pairs PBS18SF (5′-CGCGCAACTTACCCACTCTC-3′) and PBS18SR (5′-ATTCCATGCAAGACTTTTCAGGC-3′) [13]. Polymerase chain reactions (PCRs) for the 18S rDNA gene and ITS region were performed in a final volume of 30 μL, containing 1xPCR buffer (Fermentas), 1.5 mM MgCl_2_, 200 μM of each dNTP, 0.5 μM of each primer, 1 U of Taq Polymerase (Fermentas) and 5 μL of template DNA. The PCRs were carried out in the Mastercycler ep gradient S (Eppendorf) using the following steps: i) ITS regions: an initial denaturation at 96 °C for 3 min, followed by 39 cycles of denaturation at 95 °C for 50 s, annealing at 52 °C for 50 s and an extension at 72 °C for 50 s, and a final elongation at 72 °C for 7 min; and ii) 18S region: an initial denaturation at 95 °C for 3 min, followed by 39 cycles of denaturation at 94 °C for 1 min, annealing at 54 °C for 45 s and an extension at 72 °C for 1 min 30 s, and a final elongation at 72 °C for 7 min. PCR products were electrophoresed on 1.5% agarose gels strained with Good View (SBS Genetech, Bratislava, Slovakia) and then purified using ExoSAP-IT™ (Amplia, Bratislava, Slovakia), following the manufacturer’s protocol. The purified PCR products were sequenced directly in both directions using the PCR primers. For sequencing of the ITS regions, one additional internal primer, ITSR3A (5′-GAGCCGAGTGATCCACC-3′) [61], was used. Sanger sequencing was carried out using a BigDye^®^ Terminator v3.1 Cycle Sequencing Kit (Applied Biosystems) and an Applied Biosystems 3130 Genetic Analyzer (Applied Biosystems). Obtained DNA sequences were assembled and edited using Sequencher software. Newly generated sequences for *Gyrodactylus* spp. were checked by the nBLAST Search Tool to assess any similarity to available congeners, then deposited in GenBank (see the species descriptions below).

The genetic variation among newly generated sequences of *Gyrodactylus* spp. was evaluated using MEGA [97]. Sequences of the 18S rDNA and ITS regions from several Eurasian *Gyrodactylus* representatives that were shown to be genetically closely related to our studied species were retrieved from the GenBank database to assess the genetic variations. This was estimated using uncorrected genetic *p-*distances in MEGA [97].

## Results

A total of 126 *Gyrodactylus* specimens were found to parasitize 124 cypriniform fish host specimens belonging to 17 species, including two catostomid and 15 leuciscid representatives (Table 1). A total of 25 *Gyrodactylus* spp. were found, ten of them considered new to science and formally described below. Our investigation further revealed *Gyrodactylus* specimens representing eight potentially new species that have apparently never been described so far. Due to their small sample sizes, which preclude proper formal descriptions, these species are simply characterized based on the morphology of haptoral sclerites, and genetic information (when available).

Herein, *Gyrodactylus* specimens were firstly identified based on their haptoral sclerites and MCO when available. Overall, differential diagnosis involving congeners, mainly from Nearctic fauna, was provided for each identified species. Descriptions of new *Gyrodactylus* spp. (see below) were supplemented by genetic data according to the delineation within *Gyrodactylus* applied by Ziȩtara and Lumme [108] and Huyse et al. [53], with ≥ 1% of intraspecific genetic variation in the ITS region regarded as an upper limit. In total, 34 and 45 ITS and 18S rDNA sequences, respectively representing 22 *Gyrodactylus* spp. were successfully obtained. The size of raw fragments generated for each marker is included in the species description sections. nBLAST queries applied to ITS and 18S rDNA fragments (accessed in September 2022) revealed either no match or a few close hits with up to 100% similarity with already published sequences (Table 2). Sequences of the ITS regions showed higher intra- and inter-species genetic variation than 18S rDNA sequences which were highly conservative (Tables S1 and S2 in Supplementary material).

**Table 2:** Summary of the nBLAST search for representative sequences of ITS regions (in bold) and 18S rDNA for available *Gyrodactylus* species related to *Gyrodactylus* species reported in the present study. *Gyrodactylus* species lacking DNA sequences or hits below 98% identity are not shown. For all species, the *E*-value was 0.0. GB AN: GenBank accession number.

*Gyrodactylus* spp.	Fish host in this study	Query cover	% Identity	Hits (by name)	Hits (by fish host)	Hits (by GB AN)	Reference
*G. atratuli*	*R. cataractae*	97%	100%	*G. colemanensis*	*S. fontinalis*	JF836090	(Gilmore et al. 2012)
		97%	100%	*Gyrodactylus* sp.	*N. crysoleucas*	KT149284	(Leis et al. 2016)
*G. colemanensis*	*C. commersonii*	**97%**	**99.6%**	*G.* ***colemanensis***	***S. fontinalis***	**JF836142**	**(Gilmore et al. 2012)**
		100%	100%	*G. colemanensis*	*S. fontinalis*	JF836090	(Gilmore et al. 2012)
		98%	100%	*Gyrodactylus* sp.	*N. crysoleucas*	KT149284	(Leis et al. 2016)
*G. ellae* n. sp.	*C. commersonii*	100%	98.6%	*G. laevisoides*	*C. eos*	KF263526	(King et al. 2013)
*G. hanseni* n. sp.	*L. chrysocephalus*	**96%**	**98.8%**	*Gyrodactylus* sp.	***N. crysoleucas***	**KT149288**	**(Leis et al. 2016)**
		100%	100%	*G. colemanensis*	*S. fontinalis*	JF836090	(Gilmore et al. 2012)
		98%	100%	*Gyrodactylus* sp.	*N. crysoleucas*	KT149284	(Leis et al. 2016)
	*S.atromaculatus*	**96%**	**99%**	*Gyrodactylus* sp.	***N. crysoleucas***	**KT149288**	**(Leis et al. 2016)**
		100%	100%	*G. colemanensis*	*S. fontinalis*	JF836090	(Gilmore et al. 2012)
		98%	100%	*Gyrodactylus* sp.	*N. crysoleucas*	KT149284	(Leis et al. 2016)
*G. huyseae* n. sp.	*L. chrysocephalus*	100%	98.6%	*G. sedelnikowi*	*B. barbatula*	AJ566378	(Matějusová et al. 2003)
		100%	98.4%	*G. carassii*	*A. alburnus*	AJ566377	(Matějusová et al. 2003)
	*N. hudsonius*	100%	98.8%	G. sedelnikowi	*B. barbatula*	AJ566378	(Matějusová et al. 2003)
		100%	98.2%	*G. carassii*	*A. alburnus*	AJ566377	(Matějusová et al. 2003)
*G. kuchtai* n. sp.	*C. neogaeus*	100%	99.5%	*G. laevisoides*	*C. eos*	KF263526	(King et al. 2013)
*G. prikrylovae* n. sp.	*P. promelas*	**47%**	**99.6%**	*Gyrodactylus* sp.	***P. promelas***	**AY099507**	**(Gilmore et al. 2012)**
		98%	98.1%	*Gyrodactylus* sp.	*N. crysoleucas*	KT149284	(Leis et al. 2016)
*G. scholzi* n. sp.	*P. promelas*	**46%**	**99.3%**	*Gyrodactylus* sp.	***P. promelas***	**AY099507**	**(Gilmore et al. 2012)**
		98%	98.1%	*Gyrodactylus* sp.	*N. crysoleucas*	KT149284	(Leis et al. 2016)
*G.* sp. “*C. neogaeus*”	*C. neogaeus*	100%	98.2%	*G. laevisoides*	*C. eos*	KF263526	(King et al. 2013)
*G.* sp. “*H. nuchalis*”	*H. nuchalis*	100%	98.8%	*G. colemanensis*	*S. fontinalis*	JF836090	(Gilmore et al. 2012)
*G.* sp. 1 “*R. atratulus*”	*R. atratulus*	100%	100%	*G. colemanensis*	*S. fontinalis*	JF836090	(Gilmore et al. 2012)
		98%	100%	*Gyrodactylus* sp.	*N. crysoleucas*	KT149284	(Leis et al. 2016)
*G.* sp. 2 “*R. atratulus*”	*R. atratulus*	100%	100%	*G. colemanensis*	*S. fontinalis*	JF836090	(Gilmore et al. 2012)
		98%	100%	*Gyrodactylus* sp.	*N. crysoleucas*	KT149284	(Leis et al. 2016)
*G. spathulatus*	*C. commersonii*	**46%**	**99.6%**	**G. *spathulatus***	***C. commersonii***	**JF836152**	**(Gilmore et al. 2012)**
		100%	99.7%	G. *spathulatus*	*C. commersonii*	JF836098	(Gilmore et al. 2012)
*G. stunkardi*	*R. atratulus*	**86%**	**98.4%**	*Gyrodactylus* sp.	***R. osculus***	**AY099508**	**(Boeger and Kritsky 2003)**
		100%	99.8%	G. *spathulatus*	*C. commersonii*	JF836098	(Gilmore et al. 2012)
*G. variabilis*	*N. crysoleucas*	**98%**	**99.6%**	*Gyrodactylus* sp.	***N. crysoleucas***	**KT149288**	**(Leis et al. 2016)**
		97%	100%	*G. colemanensis*	*S. fontinalis*	JF836090	(Gilmore et al. 2012)
		96%	100%	*Gyrodactylus* sp.	*N. crysoleucas*	KT149284	(Leis et al. 2016)

Family: Gyrodactylidae Cobbold, 1864

Genus: *Gyrodactylus* Nordmann, 1832

### *Gyrodactylus ellae* n. sp. (Fig. 1A)

urn:lsid:zoobank.org:act:0DC8BB87-6670-4FAF-A4E7-DE837D4C5AFD

**Figure 1:**
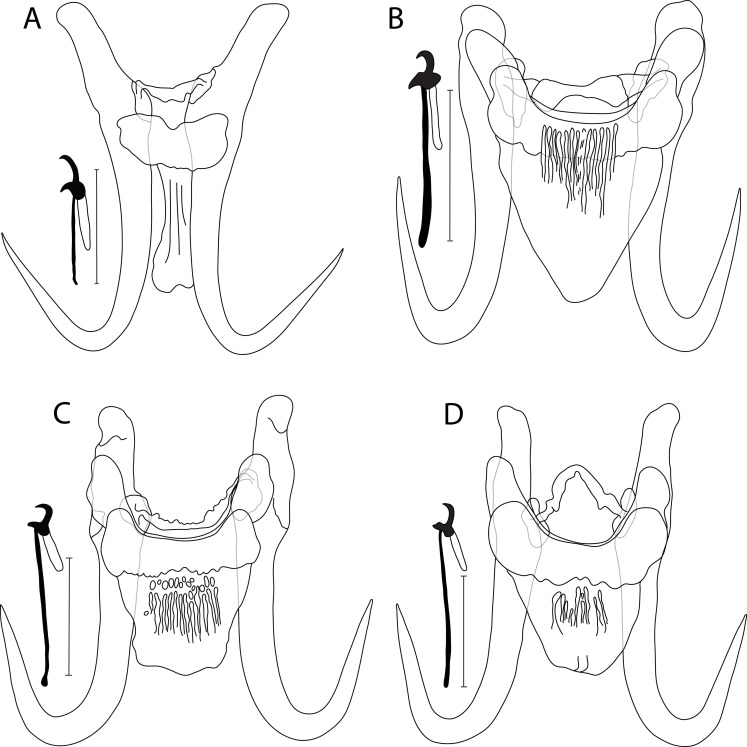
Drawing of haptoral sclerotized structures of newly described *Gyrodactylus* spp.: (A) *G. ellae* n. sp. ex *C. commersonii*; (B) *G. hamdii* n. sp. ex *C. commersonii*; (C) *G. hanseni* n. sp. ex *L. chrysocephalus*, and (D) ex *S. atromaculatus*. Scale bar = 20 μm.

Type-host: white sucker *Catostomus commersonii* (Lacepède, 1803) (Catostomidae)

Host GenBank accession number: OR269995

Site of infection: fins

Type-locality: Leatherstocking Creek, Otsego, New York, USA

Additional locality: Rom Hill Beaver Pond, Cooperstown, New York, USA

Type-material: Holotype: MNHN HEL1996, paratypes: MNHN HEL1997-1998.

Infection indices: 25%, 1–4 monogeneans per infected host

Parasite GenBank accession number: 18S rDNA: OR269952; ITS: OR269995

Etymology: the specific name honors Ella, a close friend of the first author.

#### Description

Haptor subcircular, anchor base lacking folds, total length 58.7 (54.4–60.4; *n* = 8); shaft slightly bowed, length 45.3 (43.2–46.9; *n* = 8); point curved and elongate, length 25.5 (24.1–26.5; *n* = 8); root moderately long, tapered, length 19.4 (17.5–20.5; *n* = 8). Ventral bar lacking lateral processes, total length 14.4 (11.8–17.7; *n* = 8), median width 6.10 (4.3–7.3; *n* = 8); membrane (shield) long, tongue-like, constricted anteriorly, extending posteriorly over 1/2 length of anchor shaft, enlarged proximally, length 20.6 (19.2–21.9; *n* = 8). Dorsal bar short, variably bent with attenuated ends inserted into terminal plates, total length 13.9 (10.8–17.2; *n* = 8). Marginal hooks total length 23.0 (21.8–23.9; *n* = 8); sickle foot significant with globose heel, pointy and curved toe, inconspicuous shelf; sickle proper as thick as toe base, shaft length 4.9 (3.1–5.5; *n* = 8); sickle length to shaft attachment 6.8 (3.5–8.8; *n* = 8); sickle proximal width 4.1 (3.2–4.7; *n* = 8); sickle distal width 4.3 (2.6–4.9; *n* = 8); relatively far reaching, short and weekly curved point, length 1.1 (0.7–1.6; *n* = 8); filament loop extending about 2/3 of handle length, length 10.9 (8.7–12.6; *n* = 8); handle length 16.4 (14.8–17.9; *n* = 8). MCO with 6–8 spinelets.

#### Differential diagnosis

Morphologically, *G. ellae* n. sp. (Fig. 1A) is similar to its genetically closely related *Gyrodactylus* sp. “*C. neogaeus*” found herein to parasitize the finescale dace *Chrosomus neogaeus* (Cope, 1867) (Leusciscidae) by the ventral bar membrane, which lacks lateral processes. Both species are, however, different due to (i) the relatively longer anchors in *C. neogaeus* (54.4–60.4 μm in *G. ellae* n. sp. vs. 67.7 μm in *Gyrodactylus* sp. “*C. neogaeus*”), and (ii) differently shaped marginal hooks (a significant sickle foot with a globose heel, pointy and curved toe, inconspicuous shelf, and a sickle proper as thick as toe base in *G. ellae* n. sp. vs. a disproportionate sickle foot with globose and dipped down heel, straight and triangular toe, conspicuous shelf, and a sickle proper of 1/2 the thickness of toe base in *Gyrodactylus* sp. “*C. neogaeus*”) with shorter sizes in *G. ellae* (21.8–23.9 μm in *G. ellae* n. sp. vs. 30.8 μm in *Gyrodactylus* sp. “*C. neogaeus*”). Overall, the haptoral morphology of *G. ellae* n. sp. is reminiscent of *G. dechtiari* Hanek & Fernando, 1971 known from Nearctic riffle daces of the genus *Rhinichthys* Agassiz, 1849 [53, 54, and references herein], and *G. laevisoides* King, Cone, Mackley & Bentzen, 2013 from the northern redbelly dace *Chrosomus eos* Cope, 1861 [46]. *Gyrodactylus ellae* n. sp. mainly differs from *G. dechtiari* by its (i) longer anchors (54.4–60.4 μm in *G. ellae* n. sp. vs. 47.7 μm in *G. dechtiari*), (ii) longer ventral bar membrane (19.2–21.9 μm in *G. ellae* n. sp. vs. 13.4 μm in *G. dechtiari*), and (iii) shorter marginal hooks and handle (21.8–23.9 and 14.8–17.9 μm in *G. ellae* n. sp. vs. 27.9 and 23.3 μm in *G. dechtiari*, respectively). It is distinguishable from *G. laevisoides* regarding (i) the shorter anchors in *G. laevisoides* (54.4–60.4 μm in *G. ellae* n. sp. vs. 34–38 μm in *G. laevisoides*), and (ii) the longer ventral bar membrane in *G. ellae* (19.2–21.9 μm in *G. ellae* n. sp. vs. 8–9.5 μm in *G. laevisoides*).

#### Molecular taxonomy

Fragments covering the ITS1 (346 bp), 5.8S (157 bp), ITS2 (349 bp), and 18S rDNA (449 bp) regions were successfully sequenced for three specimens of *G. ellae* nov. sp. parasitizing *C. commersonii* sampled in two close Northeastern localities (New York, USA) (Table 1). The ITS and 18S rDNA sequences obtained for this species did not show any intraspecific variation. Sequences of the ITS regions did not show any close hit to *G. ellae* nov. sp. (Tables 2 and S1). Considering haptoral morphology, *Gyrodactylus* sp. “*C. neogaeus*” identified herein was the closest hit to *G. ellae* nov. sp. based on 18S rDNA sequences (*p-*distances = 0.9%, 4 bp; Tables 2 and S2).

### *Gyrodactylus hamdii* n. sp. (Fig. 1B)

urn:lsid:zoobank.org:act:045C6973-4B10-4DC4-A7E8-0C1BEE0FE249

Type-host: white sucker *Catostomus commersonii* (Lacepède, 1803) (Catostomidae)

Host GenBank accession number: OR269996, OR269997

Site of infection: fins

Type-locality: Rom Hill Beaver Pond, Cooperstown, New York, USA

Additional locality: Leatherstocking Creek, Otsego, New York, USA; Cap-Rouge River, Quebec, Canada

Type-material: Holotype: MNHN HEL1999, paratypes: MNHN HEL2000-2001.

Infection indices: 12%, 1–2 monogeneans per infected host

Parasite GenBank accession number: 18S rDNA: OR269953, OR269954; ITS: OR269996, OR269997

Etymology: the specific name honors Hamdi Mohamed Salim, an old close friend of the first author.

#### Description

Haptor subcircular, anchor base lacking folds, total length 44.9 (43.4–46.1; *n* = 5); shaft slightly bowed, length 35.6 (34.8–37; *n* = 5); point curved and elongate, length 20.3 (17.2–23.8; *n* = 5); root short, length 11.7 (11.3–12.5; *n* = 5). Ventral bar with blunt lateral processes extending out of bar, total length 25.4 (23.1–26.6; *n* = 5), median width 5.7 (5.1–6.4; *n* = 5); distance between tips 30.5 (29.1–32.3; *n* = 5); membrane (shield) triangular, extending posteriorly almost the length of anchor shaft, tapering to a broadly rounded posterior with several fine longitudinal striations in its centre, length 16.6 (15.7–19.0; *n* = 5). Dorsal bar variably bent, constricted at midpoint with posteriorly directed projections, attenuated ends inserted into terminal plates, total length 24.3 (22.1–27.1; *n* = 5). Marginal hooks total length 25.6 (24.3–26.4; *n* = 5); sickle foot significant with ellipsoid heel, triangular toe, conspicuous shelf; sickle proper of 1/2 the thickness of toe base, shaft length 3.3 (2.7–3.9; *n* = 5); sickle length to shaft attachment 4.8 (4.3–5.2; *n* = 5); sickle proximal width 4.4 (4.0–4.7; *n* = 5); sickle distal width 2.5 (2.1–3.1; *n* = 5)_;_ short and curved point, length 1.7 (1.4–1.8; *n* = 5); filament loop extending about 1/3 of handle length, length 6.9 (6.1–8.4; *n* = 5); handle length 21.1 (19.4–21.9; *n* = 5). MCO with 6–8 spinelets.

#### Differential diagnosis

No intraspecific variation in haptoral sclerites was found on a geographical scale (Canada vs. USA). *Gyrodactylus hamdii* n. sp. (Fig. 1B) seems morphologically closely related to *G. commersoni* Threlfall, 1974 and *G. wardi* Kritsky & Mizelle, 1968, both known from a range of Nearctic suckers of the genus *Catostomus* Lesueur, 1817 [39, 49, 66, 99], and to its undescribed congener *Gyrodactylus* sp. 2 “*R. atratulus*” (see below). The new species is, however, distinguishable from *G. commersoni* by (i) its shorter anchors (43.4–46.1 μm in *G. hamdii* n. sp. vs. 56–58 μm in *G. commersoni*), (ii) a differently-shaped ventral bar membrane (triangular in *G. hamdii* n. sp. vs. oval in *G. commersoni*), and (iii) different marginal hooks (sickle foot with an ellipsoid heel, triangular toe, conspicuous shelf, and a sickle proper of 1/2 the thickness of toe base in in *G. hamdii* n. sp. vs. sickle foot with a globose heel, blunt toe lacking a shelf (according to original drawings in [99])). *Gyrodactylus hamdii* n. sp. is different from *G. wardi* by (i) its shorter anchors (43.4–46.1 μm in *G. hamdii* n. sp. vs. 57–59 μm in *G. wardi*), and (ii) shorter ventral bar (23.1–26.6 μm in *G. hamdii* n. sp. vs. 31–32 μm in *G. wardi*) (measurements of *G. wardi* available in [99]). *Gyrodactylus hamdii* n. sp. differs from *Gyrodactylus* sp. 2 “*R. atratulus*” by (i) its shorter anchors (43.4–46.1 μm in *G. hamdii* n. sp. vs. 70.2 μm in *Gyrodactylus* sp. 2 “*R. atratulus*”), (ii) shorter ventral bar membrane (15.7–19.0 μm in *G. hamdii* n. sp. vs. 26.4 μm in *Gyrodactylus* sp. 2 “*R. atratulus*”), and (iii) shorter marginal hooks (24.3–26.4 μm in *G. hamdii* n. sp. vs. 32.5 μm in *Gyrodactylus* sp. 2 “*R. atratulus*”).

#### Molecular taxonomy

Fragments covering the ITS1 (417 bp), 5.8S (157 bp), ITS2 (389 bp), and 18S rDNA (434 bp) regions were obtained from a single specimen of *G. hamdii* n. sp. parasitizing a Northeastern *C. commersonii* (New York, USA) (Table 1). From Eastern Canada catostomids (Quebec), sequences of two specimens of *G. hamdii* n. sp. (987 bp) were obtained for the ITS regions covering ITS1 (417 bp), 5.8S (157 bp), ITS2 (389 bp), and 18S rDNA (434 bp). No intraspecific variation was found in either nucleotide sequence on a geographical scale. nBLAST search based on the ITS and 18S rDNA sequences did not reveal any already published hits (Table 2). *Gyrodactylus wardi* described on the Sacramento sucker *Catostomus occidentalis* Ayres, 1854 (Catostomidae) [49] was the sole genetically closest congener to *G. hamdii* n. sp. based on the ITS region sequences (*p-*distances = 2.6%, 25 bp; Table S1).

### *Gyrodactylus hanseni* n. sp. (Figs. 1C, 1D)

urn:lsid:zoobank.org:act:637D82CD-DBCC-459B-A87F-9234B1194595

Type-host: striped shiner *Luxilus chrysocephalus* Rafinesque, 1820 (Leuciscidae)

Additional host: creek chub *Semotilus atromaculatus* (Mitchill, 1818) (Leuciscidae)

Site of infection: fins for both hosts

Host GenBank accession number: OR269998, OR269999

Type-locality: Ouachita Mountains Biological Station, Polk County, Arkansas, USA

Additional locality: Caddo River for *L. chrysocephalus* and Big Fork Creek for *S. atromaculatus*, both in Polk County, Arkansas, USA

Type-material: Holotype: MNHN HEL2002, paratypes: MNHN HEL2003-2004.

Infection indices: 12.5%, 1–12 monogeneans per infected host

Parasite GenBank accession number: 18S rDNA: OR269955, OR269956; ITS: OR269998, OR269999

Etymology: the specific name honors Dr. Haakon Hansen from the Norwegian Veterinary Institute (Oslo, Norway) for his extensive work on *Gyrodactylus* in the past years.

#### Description

Haptor subcircular, anchor base may possess folds, total length 60.3 (57.1–63.8; *n* = 15); shaft straight, length 43.7 (41.7–47.5; *n* = 15); point curved and elongate, length 25.5 (20.5–28.5; *n* = 15); root moderately long, length 19.9 (17.5–22.4; *n* = 15). Ventral bar with blunt lateral processes extending out of bar, total length 26.7 (23.4–28.5; *n* = 15), median width 7.6 (4.7–8.8; *n* = 15); distance between tips 31 (27.1–34.5; *n* = 15); membrane (shield) almost trapezoid, extending posteriorly almost 2/3 of length of anchor shaft, tapering posteriorly with several ridges and fine longitudinal striations in its centre, length 18.3 (16.3–23.4; *n* = 15). Dorsal bar variably bent, slightly to well curved, can show posteriorly directed projections, with attenuated ends inserted into terminal plates, total length 24.5 (19.6–28.6; *n* = 15). Marginal hooks total length 32 (31.2–32.9; *n* = 15); sickle foot almost oval with globose heel, blunt to triangular toe, inconspicuous to conspicuous shelf; sickle proper as thick as toe base, shaft length 3.6 (3.1–4.1; *n* = 15); sickle length to shaft attachment 4.6 (4.4–4.9; *n* = 15); sickle proximal width 3.7 (2.7–4.1; *n* = 15); sickle distal width 2.8 (2.4–3.4; *n* = 15); point relatively long and curved, length 1.6 (1.4–1.8; *n* = 15); filament loop extending about 1/3 of handle length, 8.1 (6.8–10.2; *n* = 15); handle length 27.4 (26.2–28.5; *n* = 15). MCO with 6–8 spinelets.

#### Differential diagnosis

So far, no formal descriptions of *Gyrodactylus* spp. parasitizing *L. chrysocephalus* or *S. atromaculatus* are available [11]. When comparing *G. hanseni* n. sp. (Figs. 1C and 1D) specimens from the two fish hosts, a weak variation is observed, mainly in (i) the ventral bar membrane, which has a broader ending in *G. hanseni* n. sp. from *L. chrysocephalus* compared to that from *S. atromaculatus*, (ii) the dorsal bar, which is slightly curved with an irregular wall in *G. hanseni* n. sp. from *L. chrysocephalus*, but well curved with posterior projections near each end in *G. hanseni* n. sp. from *S. atromaculatus*, and (iii) the shape of marginal hooks where the sickle foot in specimens from *L. chrysocephalus* lacks the shelf in the sickle toe, a feature present in specimens from *S. atromaculatus*. Regarding the *Gyrodactylus* sp. from captive *N. crysoleucas*, which is genetically identical to *G. hanseni* n. sp. (see below), Leis et al. [54] assumed this species (holotype of poor quality) to be *G. variabilis* Mizelle & Kritsky, 1967 [68] formally described from non-native *N. crysoleucas* (introduced in California, see [90]). The sizes of haptoral sclerites in specimens from *L. chrysocephalus* and *S. atromaculatus* considerably overlap with those in *Gyrodactylus* sp. from *N. crysoleucas.* When comparing our specimens to those of *G. variabilis*, considerable variation in the dorsal bar is observed (19.6–28.6 μm in *G. hanseni* n. sp. vs. 12–14 μm in *G. variabilis*). Leis et al. [54] reported further differences in the shape of the sickle of the marginal hooks (a more compact sickle in *Gyrodactylus* sp. from *N. crysoleucas* vs. a long and thin one in *G. variabilis*). Previous parasitological investigation of *S. atromaculatus* [11] in Eastern Canada revealed the presence of a single specimen of *Gyrodactylus* sp. So far, their study represents this host’s sole record of *Gyrodactylus* spp., but no drawings or measurements were provided. Therefore, it is impossible to state whether or not the specimen recovered by Cone [11] represents *G. hanseni* n. sp. Overall, the morphology of the haptoral sclerites of *G. hanseni* n. sp., especially that of the ventral bar, is strongly reminiscent of that of *G. asperus* Rogers, 1967 parasitizing the rough shiner *Notropis baileyi* Suttkus & Raney, 1955 [85] and of *G. lythruri* Rogers, 1975 from *Lythrurus* spp. Jordan, 1876 [86]. *Gyrodactylus hanseni* n. sp. differs from *G. asperus* by its (i) longer dorsal bar (19.6–28.6 μm in *G. hanseni* n. sp. vs. 16–18 μm in *G. asperus*), and (ii) shorter marginal hooks handle (termed shank in [85]) (26.2–28.5 μm in *G. hanseni* n. sp. vs. 27–35 μm in *G. asperus*). The newly-described species is distinguishable from *G. lythruri* by (i) its longer dorsal bar (19.6–28.6 μm in *G. hanseni* n. sp. vs. 11–16 μm in *G. lythruri*), and (ii) its relatively longer ventral bar membrane (16.3–23.4 μm in *G. hanseni* n. sp. vs. 13–17 μm in *G. lythruri*).

#### Molecular taxonomy

Fragments covering the ITS1 (370 bp), 5.8S (157 bp), and ITS2 (389 bp) regions, as well as 18S rDNA (439 bp) were successfully sequenced for two specimens from each of *S. atromaculatus* and *L. chrysocephalus*, respectively both sampled from South-central localities (Arkansas, USA) (Table 1). nBLAST search using ITS sequences retrieved *Gyrodactylus* sp. (KT149288) from *N. crysoleucas* [54] as the closest hit with respect to our specimens (Table 2). On the basis of the ITS dataset and the limit for species delineation [41, 108], DNA sequences from *G. hanseni* n. sp. specimens parasitizing two leuciscid hosts belonged to a single species with low intraspecific variation. However, with respect to ITS sequences, genetic variation between *G. hanseni* n. sp. and *G. variabilis*, both from *N*. *crysoleucas* was weak (*p-*distances = 1–1.3%, 9–12 bp; Table S1). The 18S rDNA sequence from *G. hanseni* n. sp. representatives was identical to that of an already published sequence from *G. colemanensis* from the salmonid *S. fontinalis* (Mitchill, 1814) [31], as well as to the newly generated one from *G. colemanensis*, the sequences obtained from undescribed *Gyrodactylus* sp. 1 “*R. atratulus*”, *Gyrodactylus* sp. 2 “*R. atratulus*” (see below), and that from *Gyrodactylus* sp. (KT149284) from *N*. *crysoleucas* [54] (Table S2).

### *Gyrodactylus huyseae* n. sp. (Fig. 2A, 2B)

urn:lsid:zoobank.org:act:5E9B7D94-1561-4C07-974C-F908FCB1BEF4

**Figure 2:**
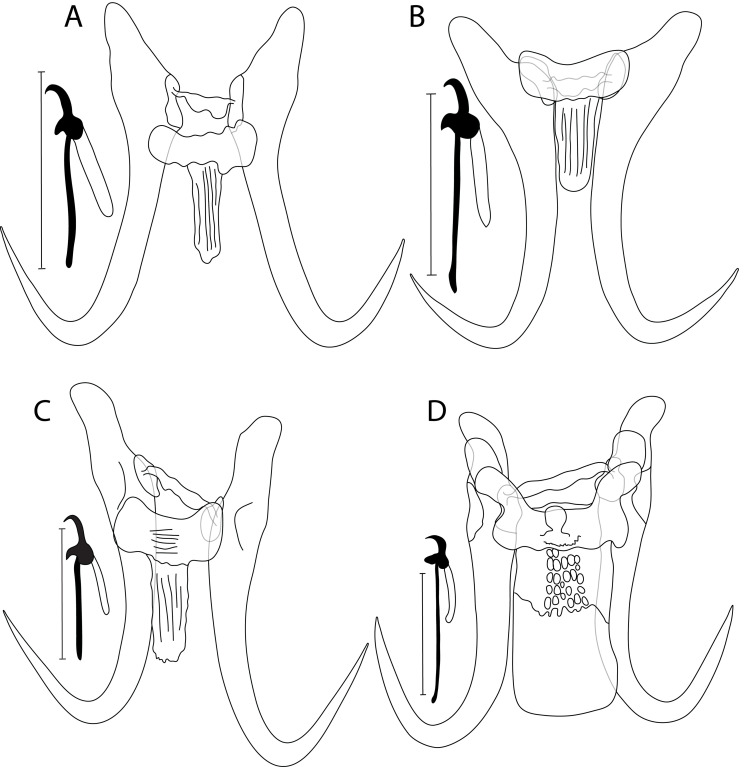
Drawing of haptoral sclerotized structures of newly described *Gyrodactylus* spp.: (A) *G. huyseae* n. sp. ex *L. chrysocephalus*, and (B) ex *N. hudsonius*; (C) *Gyrodactylus kuchtai* n. sp. ex *C. neogaeus*; and (D) *G. lummei* n. sp. ex *C. spadiceum*. Scale bar = 20 μm.

Type-host: striped shiner *Luxilus chrysocephalus* Rafinesque, 1820 (Leuciscidae)

Additional host: spottail shiner *Notropis hudsonius* (Clinton, 1824) (Leuciscidae)

Host GenBank accession number: OR270000, OR270001

Site of infection: fins and gills of *L. chrysocephalus,* fins of *N. hudsonius*

Type-locality: Ouachita Mountains Biological Station, Polk County, Arkansas, USA

Additional locality: Reed Creek for *L. chrysocephalus*, Polk County, Arkansas; Rom Hill Beaver Pond for *N. hudsonius*, Cooperstown, New York, USA

Type-material: Holotype: MNHN HEL2005, paratypes: MNHN HEL2006-2007.

Infection indices: 17.6%, 1–2 monogeneans per infected host

Parasite GenBank accession number: 18S rDNA: OR269957, OR269958; ITS: OR270000, OR270001

Etymology: the specific name honors Dr. Tine Huyse from the Royal Museum for Central Africa (Tervuren, Belgium) in recognition of her crucial work on *Gyrodactylus* genetics.

#### Description

Haptor subcircular, anchor base lacking folds, total length 34.6 (32.6–36.4; *n* = 4); shaft slightly bowed, length 27.9 (25.6–29.1; *n* = 4); point curved and elongate, length 13.6 (12.4–14.4; *n* = 4); root short, length 12 (10.5–13.5; *n* = 4). Ventral bar lacking lateral processes, total length 11.8 (10.9–12.9; *n* = 4), median width 3.5 (3.2–3.8; *n* = 4); membrane (shield) narrow, spine-like, extending proximally about 1/2 length of anchor shaft with fine longitudinal striations, length 11.2 (10.4–12.9; *n* = 4). Dorsal bar variably bent, constricted at midpoint with attenuated ends inserted into terminal plates, total length 10 (8.1–11.6; *n* = 4). Marginal hooks total length 21.9 (20.5–24.5; *n* = 4); sickle of variable shape, most common shape with significant foot, globose heel, relatively short triangular toe with conspicuous shelf; sickle proper almost as thick as toe base, shaft length 4.0 (3.6–4.3; *n* = 4); sickle length to shaft attachment 5.1 (4.7–5.6; *n* = 4); sickle proximal width 3.8 (3.3–4.1; *n* = 4); sickle distal width 3.4 (2.9–3.8; *n* = 4); relatively short and curved point, length 1.7 (1.5–1.9; *n* = 4); filament loop extending about 2/3 of handle, length 8.6 (7.6–9.4; *n* = 4); handle length 17 (15.7–19.1; *n* = 4). MCO not observed.

#### Differential diagnosis

Herein, *N. hudsonius* has been investigated for *Gyrodactylus* spp. for the first time. Morphologically, *G. huyseae* n. sp. specimens from *L. chrysocephalus* and those from *N. hudsonius* did not show any obvious variation in their haptoral sclerites. *Gyrodactylus huyseae* n. sp. (Figs. 2A and 2B) can be compared to *G. baeacanthus* from the blacktail shiner *Cyprinella venusta* Girard, 1856 (Leuscicidae) [103] and the comely shiner *N. amoenus* [49], *G. dechtiari* [32], and *G. laevisoides* [46] regarding the overall morphology of their haptoral sclerites. However, the new species differs from *G. baeacanthus* mainly by the shape of the dorsal bar (constricted at the midpoint in *G. huyseae* n. sp. vs. straight and vacuolated in *G. baeacanthus*). *Gyrodactylus huyseae* n. sp. is distinguishable from *G. dechtiari* regarding its shorter anchors (33.4–36.4 μm in *G. huyseae* n. sp. vs. 45 μm in *G. dechtiari*). It is different from *G. laevisoides* by (i) its relatively longer marginal hooks (20.5–24.5 μm in *G. huyseae* n. sp. vs. 17–19 μm in *G. laevisoides*), and (ii) the differently shaped ventral bar membrane (constricted distally in *G. huyseae* n. sp. vs. rectangular and distally rounded in *G. laevisoides* (visible in the original drawing, but not mentioned in the species description)).

#### Molecular taxonomy

Fragments covering ITS1 (349 bp), 5.8S (157 bp), ITS2 (350 bp), and 18S rDNA (449 bp) were successfully sequenced for three parasite specimens from *L. chrysocephalus* and a single specimen parasitizing *N. hudsonius*. Monogenean specimens were recovered from Northeastern (New York, USA) and South-central (Arkansas, USA) leuciscids (Table 1). While no close match was found with nBLAST search using the ITS sequences, 18S rDNA sequences showed high genetic similarities between *G. huyseae* n. sp. specimens from each of *L. chrysocephalus* and *N. hudsonius* and Eurasian *G. carassii* Malmberg, 1957 (AJ566377) parasitizing bleak *Alburnus alburnus* (Linnaeus, 1758), and *G. sedelnikowi* Gvozdev, 1950 (AJ566378) from the stone loach *Barbatula barbatula* (Linnaeus, 1758) [60] (Table 2). Among the studied species, *G. kuchtai* n. sp. and the undescribed *Gyrodactylus* sp. “*C. neogaeus*” were genetically the closest to *G. huyseae* n. sp., based on the ITS sequences (Table S1). Sequences of the ITS regions showed a variation around the limiting value (*p*-distances = 1–1.3%, 8–11 bp; Table S1) [41, 108], while a relatively weak variation was obtained using the 18S rDNA dataset (*p*-distances = 0.2%, 1 bp; Table S2) (see the discussion part for details about this taxonomic assessment).

### *Gyrodactylus kuchtai* n. sp. (Fig. 2C)

urn:lsid:zoobank.org:act:DDBA1595-97B0-4F7E-8845-174CBABB7B77

Type-host: finescale dace *Chrosomus neogaeus* (Cope, 1867) (Leusciscidae)

Host GenBank accession number: OR270002

Site of infection: gills

Type-locality: Mink River, Door County, Wisconsin, USA

Type-material: Holotype: MNHN HEL2008; paratypes: MNHN HEL2009-2010.

Infection indices: 23.1%, 1–2 monogeneans per infected host

Parasite GenBank accession number: 18S rDNA: OR269959; ITS: OR270002

Etymology: the specific name honors Dr. Roman Kuchta from the Laboratory of Helminthology of the Institute of Parasitology of the Biology Centre, Czech Academy of Science (České Budějovice, Czech Republic) for his extensive work on parasitic helminths.

#### Description

Haptor subcircular, anchor base with conspicuous folds, total length 45 (44.3–45.9; *n* = 4); shaft slightly bowed, length 34.7 (33.7–35.9; *n* = 4); point curved and elongate, length 18.4 (18.2–18.9; *n* = 4); root moderately short, tapered, length 16.1 (15.2–16.8; *n* = 4). Ventral bar lacking lateral processes, total length 15.8 (13.8–17.9; *n* = 4), median width 6.1 (5.7–6.9; *n* = 4); membrane (shield) narrow, spine–like, extending proximally about 1/2 length of anchor shaft, length 13.5 (12.9–14.4; *n* = 4). Dorsal bar variably bent, with attenuated ends inserted into terminal plates, total length 14.1 (12.8–15.9; *n* = 4). Marginal hooks total length 20.3 (19.9–20.8; *n* = 4); sickle foot significant with globose heel, curved and pointed toe, moderately conspicuous shelf; sickle proper of 1/2 the thickness of toe base, shaft length 4.2 (3.4–4.8; *n* = 4); sickle length to shaft attachment 5.1 (4.7–5.6; *n* = 4); sickle proximal width 4.3 (4.2–4.4; *n* = 4); sickle distal width 4.8 (4.6–5.2; *n* = 4); thin and curved point, length 1.3 (1.2–1.5; *n* = 4); filament loop extending about 2/3 of handle length, length 10 (8.9–11.8; *n* = 4); handle length 13.7 (13.4–14.1; *n* = 4). MCO not observed.

#### Differential diagnosis

*Gyrodactylus kuchtai* n. sp. (Fig. 2C) and its close sibling *G. laevisoides* show highly similar overall haptoral morphology. However, obvious differentiation is observed in the anchor size (44.3–45.9 μm in *G. kuchtai* n. sp. vs. 34–38 μm in *G. laevisoides*). The marginal hooks of *G. kuchtai* n. sp. and *G. ellae* n. sp. described above possess a similar shape and size. *Gyrodactylus kuchtai* n. sp. shares Leuciscid hosts with *G. nebraskensis* Mayes, 1977; however, *G. kuchtai* n. sp. is differentiated from this species by (i) its thinner ventral bar membrane, and (ii) a dorsal bar lacking projections near each end (based on original drawings in [65]). *Gyrodactylus kuchtai* n. sp. is distinguishable from *G. ellae* n. sp. regarding its shorter anchors (44.3–45.9 μm in *G. kuchtai* n. sp. vs. 54.4–60.4 μm in *G. ellae* n. sp.), and from the undescribed *Gyrodactylus* sp. “*C. neogaeus*” by (i) its shorter anchors (44.3–45.9 μm in *G. kuchtai* vs. 67.7 μm in *Gyrodactylus* sp. “*C. neogaeus*”), and (ii) the very typical shape of marginal hooks with a relatively longer handle in *Gyrodactylus* sp. “*C. neogaeus*” (13.4–14.1 μm in *G. kuchtai* n. sp. vs. 20.6 μm in *Gyrodactylus* sp. “*C. neogaeus*”).

#### Molecular taxonomy

Fragments covering ITS1 (355 bp), 5.8S (157 bp), ITS2 (350 bp), and 18S rDNA (449 bp) were successfully sequenced for two *G. kuchtai* n. sp. specimens from *C. neogaeus* inhabiting a Midwest location (Wisconsin, USA) (Table 1). No close relative was found based on sequences of the ITS regions (Tables 2 and S1), whereas nBLAST search based on the 18S rDNA sequences yielded the recovery of *G. laevisoides* (KF263526) from *C. eos* [46] as the closest known hit (Tables 2 and S2).

### *Gyrodactylus lummei* n. sp. (Fig. 2D)

urn:lsid:zoobank.org:act:9113D6F6-30F3-4F69-A1BD-624A1B28A583

Type-host: highland stoneroller *Campostoma spadiceum* (Girard, 1856) (Leuciscidae)

Host GenBank accession number: OR270003

Site of infection: fins

Type-locality: Big Fork Creek, Polk County, Arkansas, USA

Additional locality: Caddo River and Butcherknife Creek, Polk County, Arkansas, USA

Type-material: Holotype: MNHN HEL2011, paratypes: MNHN HEL2012-2013.

Infection indices: 18.2%, 1–2 monogeneans per infected host

Parasite GenBank accession number: 18S rDNA: OR269960; ITS: OR270003

Etymology: the specific name honors Dr. Jaakko Lumme from the Biology Department at University of Oulu (Oulu, Finland) for his extensive work on *Gyrodactylus* for most of his career.

#### Description

Haptor subcircular, anchor base with conspicuous folds, total length 55.1 (53.1–57.1; *n* = 4); shaft slightly bowed, length 43.0 (42.1–44.1; *n* = 4); point curved and elongate, length 23.1 (21.7–25.1; *n* = 4); root short, length 15.2 (14.5–16.1; *n* = 4). Ventral bar with blunt lateral processes extending out of bar, total length 22.2 (21.0–22.8; *n* = 4), median part with large knob, width 5.6 (5.6–6.0; *n* = 4); distance between tips 29.3 (27.9–29.9; *n* = 4); membrane (shield) long, rectangular, plate-like extending posteriorly almost length of anchor shaft, with lateral margins and rows of ovate to rectangular ridges ending almost at halfway along membrane, length 25.3 (22.3–27.8; *n* = 4). Dorsal bar simple, slightly tilted with attenuated ends inserted into terminal plates, total length 21.5 (20.7–22.4; *n* = 4). Marginal hooks total length 33.4 (27.4–37.0; *n* = 4); sickle moderately significant, almost triangular with globose heel, thick triangular toe, inconspicuous shelf; sickle proper almost as thick as toe base, shaft length 3.9 (3.3–5.0; *n* = 4); sickle length to shaft attachment 4.8 (4.6–5.1; *n* = 4); sickle proximal width 3.7 (3.4–3.9; *n* = 4); sickle distal width 3.1 (2.7–3.4; *n* = 4); thin and slightly curved point, length 1.6 (1.4–1.8; *n* = 4); filament loop extending about 1/2 handle length, length 8.5 (8.1–9.0; *n* = 4); handle length 31.6 (31.1–32.6; *n* = 4). MCO not observed.

#### Differential diagnosis

Haptoral morphology exhibited by *G. lummei* n. sp. (Fig. 2D) resembles each of the morphologies of the undescribed *Gyrodactylus* sp. 1 “*C. spadiceum*” (Fig. 6A) and *G. campostomae* Wellborn, 1967 known from *Campostoma* species [10, 49, 102]. *Gyrodactylus lummei* n. sp. is distinguishable from the former species in having (i) shorter anchors (53.1–57.1 μm in *G. lummei* n. sp. vs. 75.4 μm in *Gyrodactylus* sp. 1 “*C. spadiceum*”), (ii) shorter marginal hooks (27.4–37 μm in *G. lummei* n. sp. vs. 59.6 μm in *Gyrodactylus* sp. 1 “*C. spadiceum*”), and (iii) a shorter handle of marginal hooks (31.1–32.6 μm in *G. lummei* n. sp. vs. 54.6 μm in *Gyrodactylus* sp. 1 “*C. spadiceum*”). *Gyrodactylus lummei* n. sp. differs from *G. campostomae* by having (i) shorter anchors (53.1–57.1 μm in *G. lummei* n. sp. vs. 74–80 μm in *G. campostomae*), (ii) a shorter ventral bar (21.0–22.8 μm in *G. lummei* n. sp. vs. 24–30 μm in *G. campostomae*), and (iii) a longer marginal hooks filament loop (21.5–32.6 μm in *G. lummei* n. sp. vs. 13–15 μm in *G. campostomae*).

#### Molecular taxonomy

Fragments covering ITS1 (369 bp), 5.8S (157 bp), ITS2 (389 bp), and 18S rDNA (439 bp) were successfully sequenced for three parasite specimens from *C*. *spadiceum* inhabiting South-central localities (Arkansas, USA) (Table 1). No intraspecific variation was found. The nBLAST search did not reveal any hit close to *G. lummei* n. sp. (Table 2) with sequences of both 18S rDNA and ITS region. Based on the morphological evidence, *Gyrodactylus* sp. 1 “*C. spadiceum*” was shown to be the closest congener to *G. lummei* n. sp. within our *Gyrodactylus* dataset based on the 18S rDNA sequences (*p*-distances = 1.2%, 5 bp; Table S2), a result not obtained with sequences of the ITS regions.

### *Gyrodactylus mendeli* n. sp. (Fig. 3A)

urn:lsid:zoobank.org:act:953494E8-AD83-4945-AC5A-EBDDC4202415

**Figure 3:**
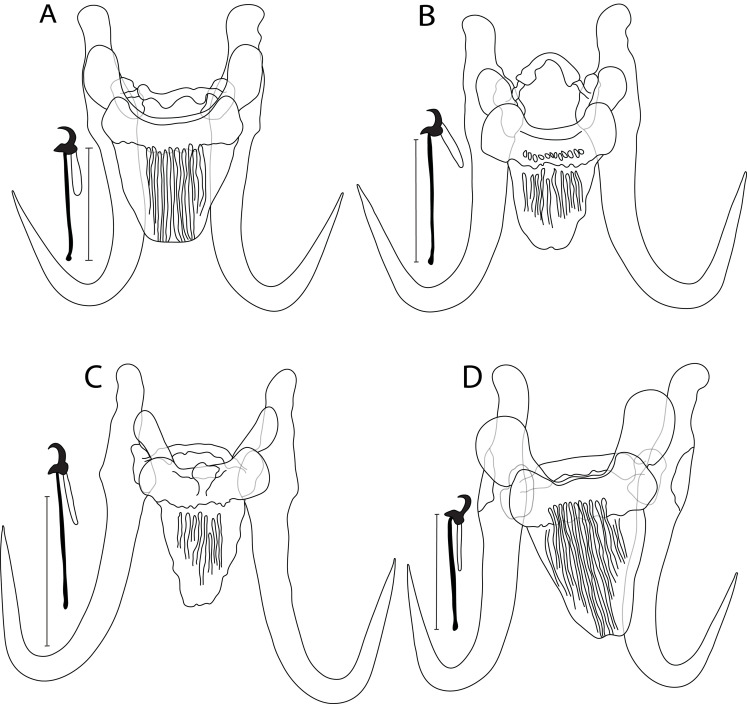
Drawing of haptoral sclerotized structures of newly described *Gyrodactylus* spp.: (A) *G. mendeli* n. sp. ex *N. biguttatus*; (B) *G. prikrylovae* n. sp. ex *P. promelas*; (C) *G. scholzi* n. sp. ex *P. promelas*; and (D) *G. steineri* n. sp. ex *N. biguttatus* (l). Scale bar = 20 μm.

Type-host: hornyhead chub *Nocomis biguttatus* (Kirtland, 1840) (Leuciscidae)

Host GenBank accession number: OR270004

Site of infection: fins

Type-locality: West Twin River, Brown County, Wisconsin, USA

Type-material: Holotype: MNHN HEL2014; paratypes: MNHN HEL2015-2016.

Infection indices: 28.6%, 1–4 monogeneans per infected host

Parasite GenBank accession number: 18S rDNA: OR269961; ITS: OR270004

Etymology: the specific name is given to honour the founder of genetics Sir Johan Gregor Mendel, who lived and worked in the city of Brno (Czech Republic).

#### Description

Haptor subcircular, anchor base may possess folds, total length 55.4 (52.2–57.7; *n* = 13); shaft slightly bowed, length 40.9 (36.2–43.7; *n* = 13); point curved and elongate, length 22.7 (16.5–25.1; *n* = 13); root moderately long, tapered, length 17.4 (14.2–22.4; *n* = 13). Ventral bar with blunt lateral processes extending out of bar, total length 25.8 (21.1–29.6; *n* = 13), median width 7 (5.7–7.8; *n* = 13); distance between tips 30.2 (27.1–36.1; *n* = 13); membrane (shield) trapezoid, extending posteriorly almost 2/3 of length of anchor shaft, tapering posteriorly with rows of longitudinal striations, length 18.5 (16.6–21.9; *n* = 13). Dorsal bar variably bent, constricted almost at halfway point with projections near each end, attenuated ends inserted into terminal plates, total length 21.9 (18.5–25.5; *n* = 13). Marginal hooks total length 26.3 (24.1–27.6; *n* = 13); sickle foot moderately significant with slightly globose heel, prominent triangular toe, conspicuous shelf; sickle proper almost as thick as toe base, shaft length 3.8 (3.1–4.4; *n* = 13); sickle length to shaft attachment 4.7 (4.3–5.3; *n* = 13); sickle proximal width 3.8 (3–4.6; *n* = 13); sickle distal width 4 (3.6–4.8; *n* = 13); point relatively long and curved, length 1.5 (1.2–1.9; *n* = 13); filament loop extending about 1/2 handle length, length 7.9 (5.8–8.9; *n* = 13); handle length 21.5 (18.6–22.6; *n* = 13). MCO with 6–8 spinelets.

#### Differential diagnosis

This is the first study reporting the presence of *Gyrodactylus* spp. on *N. biguttatus*. *Gyrodactylus mendeli* n. sp. (Fig. 3A) shows an overall morphology of haptoral sclerotized structures similar to those morphologies found in *G. asperus* from *N. baileyi* [85], *G. parvicirrus* from *Notropis atherinoides* Rafinesque, 1818 [86], and *G. planensis* Mayes, 1977 parasitizing the bigmouth shiner *Notropis dorsalis* (Agassiz, 1854) [65]. The newly described species differs from *G. asperus* [85] in having relatively shorter anchors (52.2–57.7 μm in *G. mendeli* n. sp. vs. 58–66 μm in *G. asperus*). *Gyrodactylus mendeli* n. sp. is distinguishable from *G. parvicirrus* [86] by having longer anchors (52.2–57.7 μm in *G. mendeli* n. sp. vs. 46–51 μm in *G. parvicirrus*), and from *G. planensis* [65] by the anchor root, which is shown to be curved medioventrally in *G. planensis*, a shape not observed in *G. mendeli* n. sp.

#### Molecular taxonomy

Fragments covering ITS1 (402 bp), 5.8S (157 bp), ITS2 (386 bp), and 18S rDNA (439 bp) were successfully sequenced for two *G. mendeli* n. sp. specimens from *N. biguttatus* occurring in Midwest localities (Wisconsin, USA) (Table 1). For each gene, no intraspecific variation was found. nBLAST search and *p*-distances did not reveal any hit close to *G. mendeli* n. sp. (Tables 2, S1, S2).

### *Gyrodactylus prikrylovae* n. sp. (Fig. 3B)

urn:lsid:zoobank.org:act:511BDC34-6ECC-4C08-8803-D204574716E0

Type-host: fathead minnow *Pimephales promelas* Rafinesque, 1820 (Leuciscidae)

Host GenBank accession number: OR270005, OR270006

Site of infection: fins and gills

Type-locality: Rom Hill Beaver Pond, Cooperstown, New York, USA

Additional locality: Morrys Creek, Door County, Wisconsin, USA

Type-material: Holotype: MNHN HEL2017; paratypes: MNHN HEL2018-2019 & HEL2034.

Infection indices: 30%, 1–9 monogeneans per infected host

Parasite GenBank accession number: 18S rDNA: OR269962, OR269963; ITS: OR270005, OR270006

Etymology: the specific name honours Dr. Iva Přikrylová from the Department of Biodiversity at the University of Limpopo (Sovenga, South Africa) for her extensive work on *Gyrodactylus* taxonomy.

#### Description

Haptor subcircular, anchor base may possess folds, total length 52.6 (49.4–54.1; *n* = 14); shaft slightly bowed, length 40.2 (37.5–42.6; *n* = 14); point curved and elongate, length 24.2 (21.8–26.2; *n* = 14); root moderately short, length 16.1 (14.2–17.1; *n* = 14). Ventral bar with blunt lateral processes extending out of bar, total length 22.3 (21.6–23.6; *n* = 14), median width 6.6 (5.1–7.9; *n* = 14); distance between tips 24.1 (20.5–26.9; *n* = 14); membrane (shield) semioval, extending posteriorly almost 2/3 of length of anchor shaft, tapering to an edge rounded posteriorly with several ridges and fine longitudinal striations, length 15.9 (13.5–19.1; *n* = 14). Dorsal bar strongly curved, constricted at midpoint with posteriorly directed projections, attenuated ends inserted into terminal plates, total length 19.9 (15.8–25.3; *n* = 14). Marginal hooks total length 26.4 (23.8–28.5; *n* = 14); sickle foot moderately significant with slightly globose heel, elongated and pointed toe, inconspicuous shelf; sickle proper almost as thick as toe base, shaft length 3.3 (2.9–3.5; *n* = 14); sickle length to shaft attachment 4.5 (4.1–4.8; *n* = 14); sickle proximal width 3.3 (2.1–4.1; *n* = 14); sickle distal width 2.7 (2.3–3.1; *n* = 14)_;_ relatively short and slightly curved point, length 1.5 (1.1–1.9; *n* = 14); filament loop extending about 1/3 handle length, length 7.5 (6.5–9.2; *n* = 14); handle length 22.1 (19.1–24.5; *n* = 14). MCO not observed.

#### Differential diagnosis

No morphological variation was observed for *G. prikrylovae* n. sp. (Fig. 3B) on the geographical scale. Despite the high morphological similarity with *G. scholzi* n. sp. (see below), consistent differences in haptoral sclerites were found to support the distinction between these two species. These differences are as follows: (i) in the shape of the ventral bar membrane, which presents a knob in *G. prikrylovae* n. sp., and (ii) in the dorsal bar, which is very often curved, and constricted at the midpoint with posteriorly directed projections in *G. prikrylovae* n. sp., but mostly straight in *G. scholzi* n. sp. (see below). Since no morphology was included in [31], where a *Gyrodactylus* sp. that was genetically close to *G. prikrylovae* n. sp. was reported from the same host species, the newly described species is comparable on the basis of haptoral sclerites to *G. hoffmani* Wellborn & Rogers, 1967, a species widely distributed on *P. promelas* [40, 68, 69, 103], and *G. lacustris* Mizelle & Kritsky, 1967 parasitizing the same host [24, 68]. Considerable overlap in the metrics of sclerotized structures was found in *G. prikrylovae* n. sp. and *G. hoffmani*. Yet, these two species can be distinguished from each other regarding the shape of (i) the ventral bar membrane (tapering to a rounded edge posteriorly in *G. prikrylovae* n. sp. vs. an almost rectangular one with sides tapering slightly in *G. hoffmani*), and (ii) the marginal hooks (a pointed toe in *G. prikrylovae* n. sp. vs. a blunt toe in *G. hoffmani*). *Gyrodactylus prikrylovae* n. sp. is discriminated from *G. lacustris* in having (i) shorter anchors (49.4–54.1 μm in *G. prikrylovae* n. sp. vs. 64–73 μm in *G. lacustris*), and (ii) slightly shorter marginal hooks (23.8–28.5 μm in *G. prikrylovae* n. sp. vs. 32–34 μm in *G. lacustris*).

#### Molecular taxonomy

Fragments covering ITS1 (388 bp), 5.8S (157 bp), ITS2 (392 bp), and 18S rDNA (439 bp) were successfully sequenced for a single *G. prikrylovae* n. sp. specimen parasitizing *P. promelas* from each of Northeastern and South-central regions (New York and Arkansas, USA, respectively) (Table 1). nBLAST search using sequences of the ITS regions and 18S rDNA indicated *Gyrodactylus* sp. (AY099507) from *P. promelas* sampled in Idaho (USA) [31], and *Gyrodactylus* sp. (KT149284) from captive *N*. *crysoleucas* [54] as the closest hits to *G. prikrylovae* n. sp., respectively. It should be noted that the query coverage of the published ITS sequence AY099507 was only 46%, as the ITS1 part and a portion of 5.8S were missing (Table 2). Weak intraspecific variation was found in ITS sequences on the geographical scale (Table S1). With an intraspecific variation exceeding the limit value with sequences of the ITS region (*p*-distances = 0.9% 7 bp, 1.6–1.7%, 15 bp; Table S1) and no genetic variation in 18S rDNA sequences (Table S2), *G. scholzi* n. sp. (see below) was recovered as the closest congener among the studied species.

### *Gyrodactylus scholzi* n. sp. (Fig. 3C)

urn:lsid:zoobank.org:act:0C33FCEA-E603-4AAA-BC92-C647320D6790

Type-host: fathead minnow *Pimephales promelas* Rafinesque, 1820 (Leuciscidae)

Host GenBank accession number: OR270007

Site of infection: gills

Type-locality: Rom Hill Beaver Pond, Cooperstown, New York, USA

Additional locality: Morrys Creek, Polk County, Arkansas, USA

Type-material: Holotype: MNHN HEL2020; paratypes: MNHN HEL2021-2022.

Infection indices: 40%, 1–4 monogeneans per infected host

Parasite GenBank accession number: 18S rDNA: OR269964; ITS: OR270007

Etymology: the specific name honors Prof. Tomáš Scholz from the Laboratory of Helminthology of the Institute of Parasitology of the Biology Centre, Czech Academy of Science (České Budějovice, Czech Republic) for his extensive work on parasitic helminths.

#### Description

Haptor subcircular, anchor base may possess folds, total length 53.4 (49.0–56.5; *n* = 9); shaft slightly bowed, length 40.3 (37.7–42.7; *n* = 9); point curved and elongate, length 24.3 (22.5–26.1; *n* = 9); root moderately long, tapered, length 16.6 (15.2–17.5; *n* = 9). Ventral bar with blunt lateral processes extending out of bar, total length 21.4 (18.5–23.3; *n* = 9), median part with a knob, width 6.6 (5.9–7.6; *n* = 6); distance between tips 23.6 (21.1–25.4; *n* = 9); membrane (shield) almost trapezoid, extending posteriorly almost 1/2 length of anchor shaft, tapering to a rounded edge posteriorly with several fine longitudinal striations, length 16.2 (12.1–19.4; *n* = 9). Dorsal bar relatively straight, with posteriorly directed projections, attenuated ends inserted into terminal plates, total length 18.8 (14.9–21.1; *n* = 9). Marginal hooks total length 26.4 (24.9–27.7; *n* = 9); sickle foot moderately significant with slightly globose heel, prominent triangular toe, conspicuous shelf; sickle proper as thick as toe base, shaft length 3.3 (2.8–3.7; *n* = 9); sickle length to shaft attachment 4.4 (4.2–5.1; *n* = 9); sickle proximal width 3.3 (2.1–4.1; *n* = 9); sickle distal width 2.6 (2.3–3.1; *n* = 9); point relatively long, well curved, length 1.5 (1.1–1.9; *n* = 9); filament loop extending about 1/2 handle length, length 7.8 (6.8–8.3; *n* = 9); handle length 21.8 (20.3–22.8; *n* = 9). MCO not observed.

#### Differential diagnosis

No interspecific morphological variation was observed within *G. scholzi* n. sp. (Fig. 3C) on the geographical scale. Comparison between *G. scholzi* n. sp. and its closely related *G. prikrylovae* n. sp. is detailed above. A few haptoral features supported the distinction between *G. scholzi* n. sp. and each of *G. hoffmani* and *G. lacustris*, both from *P. promelas* [68, 103]. The new species is mainly different from *G. hoffmani* by the knob in the median part of the ventral bar, a feature absent in the latter. Similarly, this structure discriminated *G. scholzi* n. sp. from *G. lacustris*, in addition to (i) shorter anchors (49.0–56.5 μm in *G. scholzi* n. sp. vs. 64–73 μm in *G. lacustris*), and (ii) slightly shorter marginal hooks (24.9–27.7 μm in *G. scholzi* n. sp. vs. 32–34 μm in *G. lacustris*).

#### Molecular taxonomy

Fragments covering ITS1 (390 bp), 5.8S (157 bp), ITS2 (392 bp), and 18S rDNA (439 bp) were successfully sequenced for two *G. scholzi* n. sp. specimens parasitizing Northeastern *P. promelas* (New York, USA) (Table 1). No intraspecific variation in the nucleotide sequences was found. nBLAST search and the calculation of genetic variation recovered identical hits as shown for *G. prikrylovae* n. sp. (see above, Tables 2, S1, S2).

### *Gyrodactylus steineri* n. sp. (Fig. 3D)

urn:lsid:zoobank.org:act:7AE69894-C5C7-4E66-B783-0A0EFCC75886

Type-host: redside dace *Clinostomus elongatus* (Kirtland, 1840) (Leuciscidae)

Host GenBank accession number: OR348748

Site of infection: fins

Type-locality: West Twin River, Brown County, Wisconsin, USA

Type-material: Holotype: MNHN HEL2023; paratypes: MNHN HEL2024.

Infection indices: 100%, 1–4 monogeneans per infected host

Etymology: the specific name honors Dr. Bohumil Steiner, the co-author’s father (M. Seifertová), who recently passed away because of the COVID-19 outbreak.

#### Description

Haptor subcircular, anchor base with conspicuous folds, total length 65.5 (63.1–67.7; *n* = 4); shaft slightly bowed, length 46.5 (42.8–50; *n* = 4); point curved and elongate, length 26.1 (25.1–28; *n* = 4); root long, tapered, length 22.7 (20.9–25; *n* = 4). Ventral bar with large, rounded blunt lateral processes extending out of bar, total length 29 (27.9–30.1; *n* = 4), median width 8.2 (6.5–10; *n* = 4); distance between tips 37.4 (35–39.4; *n* = 4); membrane (shield) trapezoid, extending posteriorly almost the length of anchor shaft, tapering to broadly rounded posterior with several fine longitudinal striations, length 22.4 (20.6–23.4; *n* = 4). Dorsal bar variably bent with projections near each end, attenuated ends inserted into terminal plates, total length 26.9 (25.6–29.2; *n* = 4). Marginal hooks total length, 28.1 (27.2–28.9; *n* = 4); sickle foot significant with globose heel, prominent triangular toe, conspicuous shelf; sickle proper of ½ the thickness of toe base, length 4 (3.9–4.1; *n* = 4); sickle length to shaft attachment 4.7 (3.8–5.5; *n* = 4); sickle proximal width 4.2 (3.8–4.8; *n* = 4); sickle distal width 4.1 (3.8–4.3; *n* = 4); point relatively thick, well curved, length 1.5 (1.3–1.8; *n* = 4); filament loop extending about 1/2 handle length, length 8 (6.5–9.4; *n* = 4); handle length 23 (22.6–23.4; *n* = 4). MCO not observed.

#### Differential diagnosis

*Gyrodactylus steineri* n. sp. (Fig. 3D) was recognized as a new and first species parasitizing *C. elongatus*. This species was formally described herein based on pertinent haptoral morphology, especially the typical shape (large and pronounced) of ventral bar lateral processes. The haptoral morphology shown by *G. steineri* n. sp. is reminiscent of that of newly described *G. huyseae* n. sp. and *G. mendeli* n. sp. from distinct leuciscid hosts (see above), as well as that of *G. asperus* from *N. baileyi* [85] and *G. parvicirrus* from *N. atherinoides* [86]. *Gyrodactylus steineri* n. sp. is distinguishable from *G. huyseae* n. sp. in having (i) longer anchors (63.1–67.7 μm in *G. steineri* n. sp. vs. 32.6–36.4 μm in *G. huyseae* n. sp.), and (ii) a longer ventral bar membrane (20.6–23.4 μm in *G. steineri* n. sp. vs. 10.4–12.9 μm in *G. huyseae* n. sp.). *Gyrodactylus steineri* n. sp. differs from *G. mendeli* n. sp. by having (i) longer anchors (63.1–67.7 μm in *G. steineri* n. sp. vs. 52.2–57.7 μm in *G. mendeli* n. sp.), and (ii) ventral bar lateral processes that are much larger and more rounded compared to those in *G. mendeli* n. sp. In addition to having this typical shape of the ventral bar, *G. steineri* n. sp. mainly differs from *G. asperus* [85] by having (i) a longer dorsal bar (25.6–29.2 μm in *G. steineri* n. sp. vs. 16–18 μm in *G. asperus*), and (ii) a shorter marginal hook handle (22.6–23.4 μm in *G. steineri* n. sp. vs. 27–35 μm in *G. asperus*). *Gyrodactylus steineri* n. sp. is discriminated from *G. parvicirrus* [86] by having (i) longer anchors (63.1–67.7 μm in *G. steineri* n. sp. vs. 46–51 μm in *G. parvicirrus*), (ii) a longer dorsal bar (25.6–29.2 μm in *G. steineri* n. sp. vs. 12–14 μm in *G. parvicirrus*), and (iii) a shorter marginal hook handle (22.6–23.4 μm in *G. steineri* n. sp. vs. 28–33 μm in *G. parvicirrus*). Efforts to generate 18S and ITS sequences for *G. steineri* n. sp. were unsuccessful.

#### New records for Nearctic *Gyrodactylus* species

Our study revealed the presence of seven *Gyrodactylus* spp. from two catostomid hosts and a single leuciscid host. Due to the small sample size for most species, no formal redescription is provided; we refer to these species as *Gyrodactylus* and characterized them with reference to DNA sequences (when available) and haptoral sclerites.

### *Gyrodactylus atratuli* Putz & Hoffman, 1963 (Fig. 4A)

Type-host and locality: blacknose dace *Rhinichthis atratulus* (Hermann, 1804) (Leuscicidae), South branch of the Leetown Run, West Virginia, USA [84]

**Figure 4:**
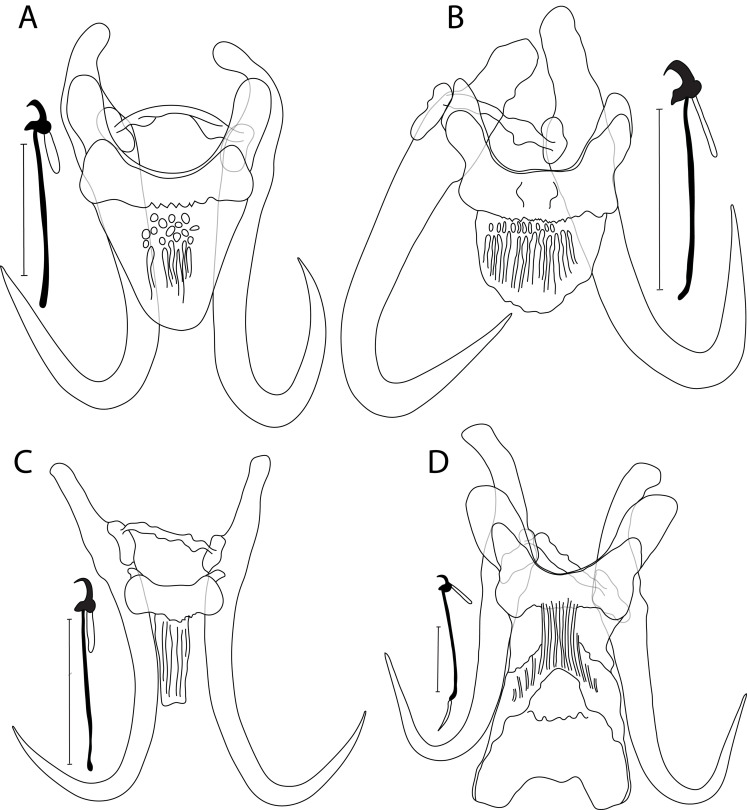
Drawing of haptoral sclerotized structures of already known *Gyrodactylus* spp.: (A) *G. atratuli* ex *R. cataractae*; (B) *G. colemanensis* ex *E. maxillingua*; (C) *G. dechtiari* ex *R. cataractae*; and (D) *G. spathulatus* ex *C. commersonii* (d). Scale bar = 20 μm.

Previous records: Allegheny pearl dace *Margariscus margarita* (Cope, 1867) (Leusciscidae), South branch of the Leetown Run, West Virginia, USA [84]; *R. atratulus* and the longnose dace *Rhinichthis cataractae* (Valenciennes, 1842) (Leusciscidae), Shelter Valley stream, Canada [32]; spotfin shiner *Cyprinella spiloptera* (Cope, 1867) (Leusciscidae), Stehman’s Run and the west branch of Little Conestoga Creek, Pennsylvania, USA [47].

Present study: *R. cataractae*, Leatherstocking Creek, Otsego, New York, USA, Cap-Rouge River, Quebec, Canada; *R. atratulus* (Hermann, 1804), Morrys Creek, Door County, Wisconsin, USA

Site of infection: fins

Voucher: MNHN HEL2025-2026.

Host GenBank accession number: OR270008

Parasite GenBank accession number: 18S rDNA: OR269965; ITS: OR270008

#### Description

Haptor subcircular, anchor base may possess folds, tips curved inward, total length 60.1 (58.2–64.4; *n* = 4); shaft slightly bowed, length 46.2 (45.3–47.3; *n* = 4); point curved and elongate, length 27.1 (25.4–27.9; *n* = 4); root relatively short, tapered, length 16.7 (14.2–21.4; *n* = 4). Ventral bar with blunt lateral processes extending out of bar, total length 26.8 (23.1–29.9; *n* = 4), median width 6.6 (5.4–8.4; *n* = 4); distance between tips 29.8 (27.8–32.2; *n* = 4); membrane (shield) trapezoid of variable size, extending posteriorly almost 2/3 of length of anchor shaft, tapering to a broadly rounded posterior with several ridges and fine longitudinal striations, length 19.7 (16.1–22.1; *n* = 4). Dorsal bar variably bent, with projections near each end, attenuated ends inserted into terminal plates, total length 22.7 (20.3–27.5; *n* = 4). Marginal hooks total length 31.9 (28.8–35.5; *n* = 4); sickle foot significant with globose heel, prominent triangular toe, conspicuous shelf; sickle proper almost as thick as toe base, shaft length 3.2 (2.3–4.1; *n* = 4); sickle length to shaft attachment 4.7 (4.6–4.9; *n* = 4); sickle proximal width 4.1 (3.8–4.2; *n* = 4); sickle distal width 2.7 (2.3–3.1; *n* = 4); point relatively thin and slightly curved, length 1.9 (1.2–2.4; *n* = 4); filament loop extending about 1/3 of handle length, length 7.3 (6.7–9.3; *n* = 4); handle length 26.7 (24.2–30.6; *n* = 4). MCO with 5–6 spinelets.

#### Differential diagnosis

The occurrence of *G. atratuli* on a range of Nearctic leusciscid hosts demonstrates its continentally wide geographic distribution and host specificity. In this study, we provided additional locality records to *G. atratuli.* Morphology of haptoral sclerites exhibited by our specimens of *G. atratuli* (Fig. 4A) and those described in [84] and identified in [32] is overall identical. Contrariwise, sizes of the sclerotized structures revealed slight intraspecific variation, mainly in (i) the anchors (58.2–64.4 μm in this study vs. 66–68 μm in [32]), (ii) the dorsal bar (20.3–27.5 μm in this study vs. 17–19 μm in [84]), and (iii) the marginal hooks (28.8–35.5 μm in this study vs. 25–28 μm in [84]). Herein, *R. atratulus* hosted, in addition to *G. atratuli*, two other distinct species that remain undescribed for lack of sufficient material (see below). Morphologically, *G. atratuli* differs from *Gyrodactylus* sp. 1 “*R. atratulus*” by the absence of a knob in the ventral bar in the former species. *Gyrodactylus atratuli* is further distinguishable from *Gyrodactylus* sp. 2 “*R. atratulus*” in having (i) a shorter ventral bar membrane (16.1–22.1 μm in *G. atratuli* vs. 37.8 μm in *Gyrodactylus* sp. 2 “*R. atratulus*”), and (ii) the lack of a filament in the handle of the marginal hooks in *G. atratuli*.

Fragments covering ITS1 (371 bp), 5.8S (157 bp), ITS2 (389 bp), and 18S rDNA (439 bp) were successfully sequenced for a single *G. atratuli* specimen parasitizing *R. R. cataractae* from eastern Canada (Quebec) (Table 1). nBLAST search (Table 2) did not indicate any close hit to *G. atratuli* using sequences of the ITS regions, while published *G. colemanensis* (JF836090) [31] and *Gyrodactylus* sp. (KT149284) [54] were found to be the closest hits based on 18S rDNA sequences. The undescribed *Gyrodactylus* sp. 2 “*R. atratulus*” inhabiting Midwest location was genetically the closest to *G. atratuli* based sequences of the ITS regions (*p*–distances = 1.7%, 16 bp; Table S1), while its 18S rDNA sequence was similar to that of a few species (Table S2).

### *Gyrodactylus colemanensis* Mizelle and Kritsky, 1967 (Fig. 4B)

Type-host and locality: rainbow trout *Oncorhynchus mykiss* (Walbaum, 1792) (Salmonidae), Coleman National Fish Hatchery, Anderson, California, USA [68]

Previous records: *O. mykiss*, Navaro River, California, USA [68]; *O. mykiss* and *S. fontinalis* (Salmonidae), Fraser Mills fish hatchery, Nova Scotia, Canada [19]; farmed *S. fontinalis*, *O. mykiss* and the Atlantic salmon *Salmo salar* Linnaeus, 1758 (Salmonidae), Nova Scotia, Canada [12]; *O. mykiss,* Coldbrook, Nova Scotia, Canada [14], and a local fish hatchery, Nova Scotia, Canada (experimental infection on captive fish) [104], both in Nova Scotia (Canada); *S. fontinalis*, *O. mykiss*, the sea trout *Salmo trutta* Linnaeus, 1758 (Salmonidae) and *S. salar*, South River watershed, Nova Scotia, Canada [107]; *O. mykiss* and *S. fontinalis*, Russia [83]; *G. colemanensis* (ITS: JF836142, 18S: JF836090), captive *S. fontinalis*, Nova Scotia, Canada [31]; *S. fontinalis,* a stream running into the Meteghan River, Nova Scotia, Canada [53].

Present study: cutlip minnow *Exoglossum maxillingua* (Lesueur, 1817) (Leuciscidae), Oaks Creek, Otsego, New York, USA

Site of infection: fins

Voucher: MNHN HEL2027.

Host GenBank accession number: OR270009

Parasite GenBank accession number: 18S rDNA: OR269966; ITS: OR270009

#### Description

Haptor subcircular, anchors not in natural position, base may possess folds, total length 46.1 (*n* = 1); shaft slightly bowed, length 34.7 (*n* = 1); point curved and elongate, length 19.5 (*n* = 1); root moderately long, length 15.0 (*n* = 1). Ventral bar with blunt lateral processes extending out of bar, total length 19.3 (*n* = 1), median width 5.7 (*n* = 1); distance between tips 22.7 (*n* = 1); membrane (shield) semioval, extending posteriorly almost 1/3 of length of anchor shaft, tapering to irregularly rounded posterior with several ridges and fine longitudinal striations, length 13.1 (*n* = 1). Dorsal bar variably bent, with projections almost near each end, attenuated ends inserted into terminal plates, total length 16.5 (*n* = 1). Marginal hooks total length 26.4 (*n* = 1); sickle foot significant and almost flat with slightly globose heel, prominent triangular toe, conspicuous shelf; sickle proper as thick as toe base, shaft length 3.5 (*n* = 1); sickle length to shaft attachment 5.2 (*n* = 1); sickle proximal width 4.3 (*n* = 1); sickle distal width 2.7 (*n* = 1); point thin and weekly curved, length 1.7 (*n* = 1); filament loop extending about 1/3 of handle length, length 6.9 (*n* = 1); handle length 21.8 (*n* = 1). MCO not observed.

#### Differential diagnosis

Morphologically, our specimens representing *G. colemanensis* (Fig. 4B) and those described by [68] overlapped considerably in terms of metrics and the shapes of hard parts. Our study can thus be considered the first one reporting the presence of *G. colemanensis* on *E. maxillingua* in Northeastern watersheds.

Fragments covering ITS1 (372 bp), 5.8S (157 bp), ITS2 (383 bp), and 18S rDNA (439 bp) were successfully sequenced for a single *G. colemanensis* specimen from Northeastern *E. maxillingua* (New York, USA) (Table 1). nBLAST search (Table 2), as well as *p*-distances using ITS sequences (Table S1) indicated high genetic similarity between our specimen and published *G. colemanensis* [31]. Sequences of 18S rDNA of these two species were identical (Table S2).

### *Gyrodactylus dechtiari* Hanek & Fernando, 1971 (Fig. 4C)

Type-host and locality: *R. atratulus*, Shelter Valley stream, Ontario, Canada [32]

Previous records: *R. cataractae,* Lake Ontario, Canada [22, 23, 32].

Present study: *R. cataractae* (Leuciscidae), Oaks Creek, Otsego, New York, USA

Site of infection: fins

Voucher: MNHN HEL2028.

Host GenBank accession number: OR270010

Parasite GenBank accession number: 18S rDNA: OR269967; ITS: OR270010

#### Description

Haptor subcircular, anchor base may possess folds, total length 47.7 (*n* = 1); shaft well bowed, length 38.8 (*n* = 1); point curved and elongate, length 17.9 (*n* = 1); root moderately long, length 15.1 (*n* = 1). Ventral bar with inconspicuous short lateral processes extending out of bar, total length 15.1 (*n* = 1), median width 5.4 (*n* = 1); distance between tips 15.1 (*n* = 1); membrane (shield) narrow, spine-like, extending proximally about 2/3 of length of anchor shaft with fine longitudinal striations, length 13.4 (*n* = 1). Dorsal bar variably bent, with projections almost near each end, attenuated ends inserted into terminal plates, total length 18.6 (*n* = 1). Marginal hooks total length 27.9 (*n* = 1), sickle foot moderately significant with slightly globose heel, thick triangular toe, conspicuous shelf; sickle proper of 1/2 the thickness of toe base, shaft length 3.5 (*n* = 1); sickle length to shaft attachment 4.6 (*n* = 1); sickle proximal width 3.2 (*n* = 1); sickle distal width 3.3 (*n* = 1); point thin, long and weekly curved, length 2.3 (*n* = 1); filament loop extending about 1/4 handle length, length 7.6 (*n* = 1); handle length 23.3 (*n* = 1). MCO not observed.

#### Differential diagnosis

*Gyrodactylus dechtiari* (Fig. 4C) is known from widely distributed *Rhinichthis* species in the Nearctic region (see previous records above). This study thus presents *R. cataractae* from Northeastern localities in the USA as a new habitat record for *G. dechtiari*. The first description of *G. dechtiari* was very brief and included a limited number of measurements and a comparison with a few, though not very morphologically similar, congeners parasitizing unrelated fish hosts [32]. Previous records of *G. dechtiari* [22, 23] did not include any morphometric characterization. Despite the small sample size herein, more detailed haptoral morphology is provided (see above). Overall, the shape and size of the haptoral sclerites exhibited by the collected specimen identified as *G. dechtiari* are identical to those included in the original description [32]. The only exception seems to be the dorsal bar, which is slightly longer in our specimen (18.6 μm vs. 13 μm in [32]), but this remains to be verified by the investigation of more specimens in the future.

A fragment covering ITS1 (421 bp), 5.8S (157 bp), ITS2 (402 bp), and 18S rDNA (437 bp) were successfully sequenced for a single *G. dechtiari* specimen inhabiting a Northeastern locality (New York, USA) (Table 1). nBLAST search did not reveal any hit close to our specimen (Table 2). The ITS sequences representing *G. dechtiari* showed considerable variation from all studied species (Table S1).

### *Gyrodactylus spathulatus* Mueller, 1936 (Fig. 4D)

Type-host and locality: *C. commersonii*, Ithaca, New York, USA [70]

Previous records: *C. commersonii,* Lake Erie, Canada [20, 24, 25], Ohio, USA [21], Lake Huron [50], Lake of the Woods and adjacent lakes, Ontario, Canada [22, 23]; silver redhorse *Moxostoma anisurum* (Rafinesque, 1820) (Catostomidae), Lake of the Woods and adjacent lakes, Canada [21]; longnose sucker *Catostomus catostomus* Rafinesque, 1820 (Catostomidae), Trout Creek, Colorado, USA [39], *C. catostomus,* Labrador, Canada [99]; *C. Catostomus*, Kolyma River, Russia [82]; *G. spathulatus* and *C. commersonii,* Nova Scotia, Canada [31].

Present study: *C. commersonii*, Rom Hill Beaver Pond, Cooperstown, and Leatherstocking Creek, Otsego, both in New York, USA

Site of infection: fins

Voucher: MNHN HEL2029.

Host GenBank accession number: OR270011

Parasite GenBank accession number: 18S rDNA: OR269968; ITS: OR270011

#### Description

Haptor subcircular, anchor base with conspicuous folds, tips curved outward, total length 106.1 (101.5–110.5, *n* = 2); shaft slightly bowed, length 75.8 (74.1–77.6, *n* = 2); point curved and elongate, length 35.8 (35.1–36.4, *n* = 2); root relatively long, length 38.3 (34.2–42.4, *n* = 2). Ventral bar with blunt lateral processes extending out of bar, total length 50.3 (40.5–60.1, *n* = 2), median width 11.7 (10.8–12.6, *n* = 2); distance between tips 63.1 (62.8–63.2, *n* = 2); membrane (shield) large, plate- or shovel-like lying between anchors, extending along length of anchor shaft with fine medial longitudinal striations, length 64.8 (63.8–65.8, *n* = 2). Dorsal bar variably bent, tilted, constricted at midpoint with attenuated ends inserted into terminal plates, with projections almost near each end, attenuated ends inserted into terminal plates, total length 33.6 (24.5–42.8, *n* = 2). Marginal hooks relatively small, total length 47.6 (37.1–58.2, *n* = 2); sickle foot almost oval and flat with semioval heel, triangular toe, inconspicuous shelf; sickle proper of 1/2 thickness of toe base, shaft length 4.1 (4.0–4.2, *n* = 2); sickle length to shaft attachment 5.7 (5.6–5.7, *n* = 2); sickle proximal width 4.6 (4.5–4.6, *n* = 2); sickle distal width 3.7 (3.7–3.8, *n* = 2); point relatively long, thin and weekly curved, length 1.3 (0.9–1.7, *n* = 2); filament loop relatively short, extending over 1/4 of handle length, length 9.7 (9.4–10.1, *n* = 2); handle ending with a filament in its posterior part, length 41.7 (30.6–52.7, *n* = 2). MCO not observed.

#### Differential diagnosis

The haptoral morphology exhibited by *G. spathulatus* (Fig. 4D) in our study was in accordance with that in the original description of Mueller [71]. This provides a new locality for *G. spathulatus* parasitizing Northeastern *C. commersonii*. Since the morphology of *G. spathulatus* was presented only in [71] (no morphological characterization of sclerotized structures was provided in [31]), our specimens are compared with those of [71]. Although Mueller [71] provided clear drawings, he supplemented them with very limited measurements of the haptoral sclerites, including the lengths of the anchors and marginal hooks only. We added the above detailed measurements for *G. spathulatus*. Overall, the haptoral sclerites of the examined specimens exhibit similar shapes regarding the sclerotized structures when compared to those of specimens included in [71]. The only difference is in terms of size, the parasite anchors in this study appear shorter than those in [71] (106.1 μm vs. 120 μm, respectively).

Fragments covering ITS1 (369 bp), 5.8S (157 bp), ITS2 (396 bp), and 18S rDNA (439 bp) were successfully sequenced for a single *G. spathulatus* specimen from Northeastern *C. commersonii* (New York, USA) (Table 1). nBLAST search based on the ITS and 18S rDNA sequences revealed high similarity, but with weak variation, between our specimen and already published *G. spathulatus* (JF836152 and JF836098) [31]. It should be noted that the coverage of the published ITS sequence was only 46%, as the ITS1 part and a portion of 5.8S were not previously sequenced (Table 2). Thus, the published ITS sequence for *G. spathulatus* was not included in the genetic variation calculation (Table S1). Based on the ITS regions and 18S rDNA sequences, *G. stunkardi* Kritsky & Mizelle, 1968, known from a range of distant hosts (see below), appeared genetically the closest to *G. spathulatus*, yet with sufficient variation (Table S1 and S2).

### *Gyrodactylus stunkardi* Kritsky & Mizelle, 1968 (Fig. 5A)

Type-host and locality: *C. occidentalis*, the Salinus River, California, USA [49]

**Figure 5:**
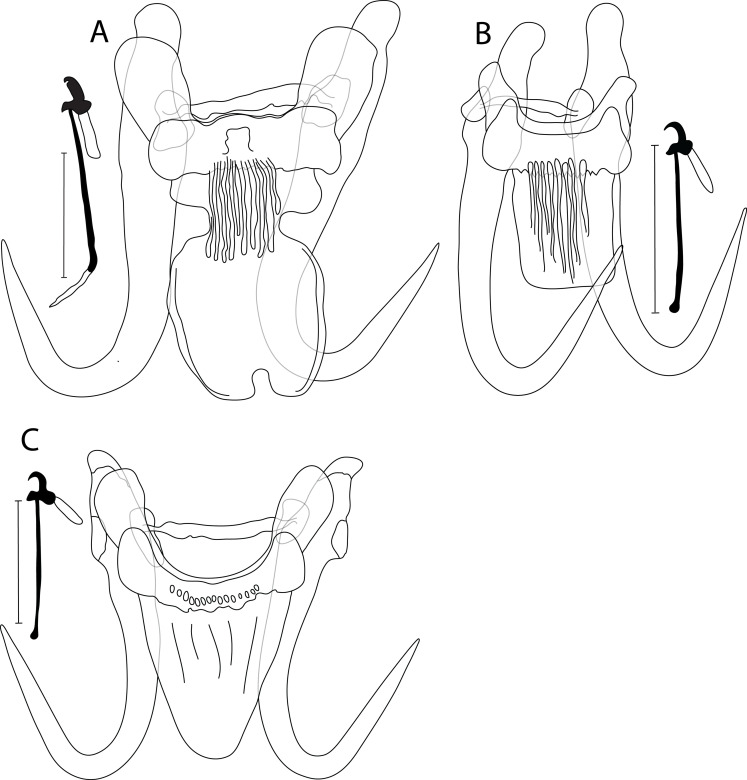
Drawing of haptoral sclerotized structures of already known *Gyrodactylus* spp.: (A) *G. stunkardi* ex *R. cataractae*; (B) *G. variabilis* ex *N. crysoleucas*; and (C) *G. wardi* ex *C. catostomus*. Scale bar = 20 μm.

Previous records: *R. atratulus* and *R. cataractae*, the Bay of Quinte, Ontario, Canada [32]; *R. cataractae*, Lake Huron, Canada [22], Grand Lake, Labrador, USA [99]; Johnny darter *Etheostoma nigrum* Rafinesque, 1820 (Percidae), Lake Huron, Canada [23]; *Gyrodactylus* sp. (ITS: AY099508), speckled dace *Rhinichthys osculus* (Girard, 1856) (Leusciscidae), Snake River, Idaho, USA [48].

Present study: *R. cataractae*, Oaks Creek, Otsego, New York and Morrys Creek, Door County, Wisconsin, USA

Site of infection: fins

Voucher: MNHN HEL2030-2031.

Host GenBank accession number: OR270012, OR270013

Parasite GenBank accession number: 18S rDNA: OR269969, OR269970; ITS: OR270012, OR270013

#### Description

Haptor subcircular, anchor base lacking folds, total length 63.1 (60.4–64.6, *n* = 8); shaft relatively straight, length 47.4 (45.4–48.7, *n* = 8); point curved and elongate, length 28.6 (27.5–29.5, *n* = 8); root moderately short, length 18.7 (17.5–19.9, *n* = 8). Ventral bar with blunt lateral processes extending out of bar, total length 27.7 (26.8–28.6, *n* = 8), median part with a knob, width 7.7 (6.5–8.5, *n* = 8); distance between tips 36.5 (34.4–38.4, *n* = 8); membrane (shield) large, plate- or shovel-like lying between anchors, extending about the same length as the anchor shaft with moderate excavation posteriorly, constricted medially with longitudinal striations, length 38.6 (37.4–39.7, *n* = 8). Dorsal bar variably bent with projections almost near each ending, attenuated ends inserted into terminal plates, total length 26.9 (25.1–28.6, *n* = 8). Marginal hooks total length 31.7 (30.1–33.3, *n* = 8), sickle foot significant but disproportionate with well-developed and globose heel, triangular and pointed toe, prominent shelf; sickle proper thick, swollen with constriction near point insertion, shaft length 4.5 (3.9–5.1, *n* = 8); sickle length to shaft attachment 5.4 (4.9–6.1, *n* = 8); sickle proximal width 5.1 (3.9–5.1, *n* = 8); sickle distal width 3.7 (3.2–4.2, *n* = 2); point reduced and thick, length 1.7 (1.3–1.9, *n* = 8); filament loop extending about 1/3 of handle length, length 8.2 (6.9–9.9, *n* = 8); handle ending with a filament in its posterior part, length 26.6 (25.9–27.6, *n* = 8). MCO with 4–6 spinelets.

#### Differential diagnosis

The shape of haptoral sclerites exhibited by our specimens of *G. stunkardi* (Fig. 5A) overlapped with that of those described by [49] and no particular variation was observed. The present study thus extends the geographical distributional range of *G. stunkardi* on the continental scale.

Fragments covering ITS1 (368 bp), 5.8S (157 bp), ITS2 (398 bp), and 18S rDNA (439 bp) were successfully sequenced for two *G. stunkardi* specimens from *R. atratulus* sampled in Northeastern location (New York, USA) and for a single parasite specimen found to parasitize South-central fish hosts (Wisconsin, USA) (Table 1). No intraspecific genetic variation was found on the geographical scale. nBLAST search (Table 2) and *p*–distances based on the ITS sequences (Table S1) revealed high genetic similarity between our specimens of *G. stunkardi* and *Gyrodactylus* sp. (AY099508) collected from *R. osculus* [48], with a variation below the limiting value (*p*-distances = 0.7%, 6 bp; Table S1). As stated above, the ITS sequences further revealed *G. spathulatus* as the closest congener to *G. stunkardi* (Table S1). Likewise, nBLAST search (Table 2) and *p-*distances based on 18S rDNA sequences (Table S2) revealed *G. spathulatus* (JF836098) from *C. commersonii* [31] as genetically the closest to *G. stunkardi* (*p-*distances = 1.2%, 5 bp).

### *Gyrodactylus variabilis* Mizelle & Kritsky, 1967 (Fig. 5B)

Type-host and locality: introduced golden shiner *Notemigonus crysoleucas*, Rooney Pond in California, USA [68]

Previous records: *Gyrodactylus* sp. (KT149288), captive *N. crysoleucas*, Minnesota, USA [54].

Present study: *N. crysoleucas*, Rom Hill Bever Pond, New York, USA, Roark Creek, Polk County, Arkansas, USA, Saint-Augustine Lake, Quebec, Canada

Site of infection: fins

Voucher: MNHN HEL2032.

Host GenBank accession number: OR270014

Parasite GenBank accession number: 18S rDNA: OR269971; ITS: OR270014

#### Description

Haptor subcircular, anchors not in natural position, base may possess folds, total length 49.7 (42.6–57.4, *n* = 3); shaft slightly bowed, length 38.1 (34.8–43.2, *n* = 3); point curved and elongate, length 22.3 (21.8–22.7, *n* = 3); root moderately long, length 14.1 (12.1–15.9, *n* = 3). Ventral bar with blunt lateral processes extending out of bar, total length 20.1 (18.4–22.8, *n* = 3), median width 5.4 (4.7–6.7, *n* = 3); distance between tips 23.4 (18.7–30.3, *n* = 3); membrane (shield) rectangular, extending posteriorly about 1/2 length of anchor shaft, slightly rounded posteriorly with fine longitudinal striations, length 14.4 (13.1–16.2, *n* = 3). Dorsal bar variably bent, with projections almost near each ending, attenuated ends inserted into terminal plates, total length 19.1 (15.1–24.7, *n* = 2). Marginal hooks total length 26.7 (24.3–31.7, *n* = 3); sickle moderately significant with globose heel, triangular toe, conspicuous shelf; sickle proper as thick as toe base, shaft length 3.1 (2.8–3.2, *n* = 2); sickle length to shaft attachment 4.3 (3.9–4.6, *n* = 3); sickle proximal width 3.6 (3.1–4.1, *n* = 2); sickle distal width 2.7 (2.4–2.9, *n* = 3); point relatively moderately thick and curved, length 1.9 (1.8–2.1, *n* = 3); filament loop extending about 1/3 of handle length, length 7.9 (7.2–8.4, *n* = 2); handle length 22.4 (19.5–27.3, *n* = 2). MCO not observed.

#### Differential diagnosis

*Gyrodactylus variabilis* (Fig. 5B) is already known from *N. crysoleucas*, but from Western localities in the USA, and on the same host in California, which represents an alien fish in this region [68]. This means that our study extends the geographical range of *G. variabilis* to Northeastern and South-central USA and Canada. Regardless of the sample size, the metrics of the haptoral sclerites, mainly the anchors, in specimens sampled in USA were closer to those obtained from specimens of the original description of *G. variabilis* [68] than to those obtained herein from Canadian fish hosts. Additionally to *G. variabilis*, *N. crysoleucas* is known to host *G. crysoleucas* Mizelle and Kritsky, 1967 [68], *G. rachelae* Price and McMahon, 1967 [81] and *G. wellborni* Nowlin, 1968 [74]. Previously, many *Gyrodactylus* spp. were recognized as a cause of gyrodactylosis in *N. crysoleucas* farms, but none of these species, including *G. variabilis*, was recognized since they were mostly misidentified at that time (see, for instance, [92]). Later on, there were a few records of *Gyrodactylus* sp. on wild-caught *N. crysoleucas* occurring in various freshwater habitats in Ontario [21, 31], and Nova Scotia [27] (both in Canada). These field studies did not investigate the parasite haptoral morphology, which makes it hard to know whether *G. variabilis* was one of the collected species.

Fragments covering ITS1 (370 bp), 5.8S (157 bp), ITS2 (389 bp), and 18S rDNA (439 bp) were successfully sequenced for a single *G. variabilis* specimen from Northeastern *N. crysoleucas* (Quebec, Canada) (Table 1). nBLAST search considering the ITS sequences revealed *Gyrodactylus* sp. (KT149288) [54] as the closest hit to our specimen, whereas published *G. colemanensis* (JF836090) [31] and *Gyrodactylus* sp. (KT149284) [54] were found to be the closest hits to *G. variabilis* based on the 18S rDNA sequences (Table 2). As previously stated, newly-described *G. hanseni* n. sp. from *L. chrysocephalus* and *S. atromaculatus* (see above) were shown to be genetically the closest to *G. variabilis* based on the ITS sequences, with a variation around the limiting value (*p-*distances = 0.9–1.3%, 7–12 bp; Table S1).

### *Gyrodactylus wardi* Kritsky & Mizelle, 1968 (Fig. 5C)

Type-host and locality: *C. occidentalis*, Salinus River, California, USA [49]

Previous records: *C. catostomus*, Trout Creek, Colorado, USA [39]; *G. atratuli*, *C. Catostomus*, Grand Lake, Labrador, USA (Threlfall [99] though it was later changed to *G. wardi* in [66]); mountain sucker *Catostomus platyrhynchus* (Cope, 1874) (Catostomidae), Utah sucker *Catostomus ardens* Jordan & Gilbert, 1881 (Catostomidae), the Cub River, Idaho, USA [66].

Present study: *C. catostomus*, Cap-Rouge River, Quebec, Canada

Site of infection: fins

Voucher: MNHN HEL2033.

Host GenBank accession number: OR270015

Parasite GenBank accession number: 18S rDNA: OR269972; ITS: OR270015

#### Description

Haptor subcircular, anchor base with conspicuous folds, tips slightly curved outward, total length 54.5 (54–55; *n* = 2); shaft slightly bowed, length 43.8 (43.9–44.1; *n* = 2); point curved and elongate, length 28.4 (28.9–30; *n* = 2); root short, length 15.7 (15.3–16; *n* = 2). Ventral bar with blunt lateral processes extending out of bar, total length 29.7 (29–30.4; *n* = 2), median width 6.9 (6.8–7; *n* = 2); distance between tips 38.7 (38.5–39; *n* = 2); membrane (shield) extending posteriorly almost the whole length of anchor shaft, tapering to a broadly rounded posterior lacking longitudinal striations, length 22.8 (22–23.7; *n* = 2). Dorsal bar straight with constricted, attenuated ends inserted into terminal plates, total length 27 (26–28; *n* = 2). Marginal hooks total length 24.8 (24–25.7; *n* = 2); sickle significant but disproportionate with well-developed globose heel, triangular toe, prominent shelf; sickle proper as thick as toe base, shaft length 2.6 (2.2–3.1; *n* = 2); sickle length to shaft attachment 3 (2.8–3.2; *n* = 2); sickle proximal width 4.1 (3.8–4.4; *n* = 2); sickle distal width 2.4 (2.1–2.8; *n* = 2); relatively thick and curved point, length 1.9 (1.7–2.1; *n* = 2); filament loop extending about 1/4 of handle length, length 4.6 (4–5.3; *n* = 2); handle length 20.9 (20–21.8; *n* = 2). MCO not observed.

#### Differential diagnosis

Overall, *G. wardi* (Fig. 5C) specimens studied herein exhibited similar haptoral morphology to that of previous records, all collected from a range of *Catostomus* spp. Yet, the sclerotized structures were shown to vary slightly at host species level (see measurements in [49, 66, 99]). The haptoral morphology exhibited by *G. wardi* is reminiscent of that of the newly described *G. hamdii* n. sp. The main differences in the sclerotized structures that allow these two species to be distinguished are in the size of anchors and ventral bars (see above in the differential diagnosis of *G. hamdii* n. sp.).

Fragments covering ITS1 (415 bp), 5.8S (157 bp), ITS2 (389 bp), and 18S rDNA (434 bp) were successfully sequenced for a single *G. wardi* specimen from Northeastern of *C. Catostomus* (Quebec, Canada) (Table 1). nBLAST search did not reveal any close hit to *G. wardi* (Table 2), while *G. hamdii* n. sp. was again shown to be the closest congener based on the ITS region (see above).

## Discussion

The investigation of viviparous gyrodactylids (Gyrodactylidae) parasitizing cypriniform fish hosts from distant Nearctic watersheds provided a good opportunity to assess the morphological and genetic diversity of these ectoparasites on a large continental scale. So far, gyrodactylids are the second largest monogenean family parasitizing Nearctic cypriniforms, with 54 *Gyrodactylus* spp. [51]. Our survey focused on a range of cypriniform fish species, including 15 and two species of Leuciscidae and Catostomidae, respectively collected from various lakes and rivers associated with distinct drainage systems in the USA and Canada. Overall, 25 *Gyrodactylus* spp. were identified, of which 18 have never previously been described. A total of 10 species were newly described herein based on a combination of morphological traits (haptoral sclerites) and genetics (sequences of the ITS regions and 18S rDNA) except for *G. steineri* n. sp. (see above). Two *Gyrodactylus* spp., specifically *G. ellae* n. sp. and *G. hamdii* n. sp., were described from the widely distributed catostomid *C. commersonii*. Similarly, two new *Gyrodactylus* spp. were described from each of the following two leuciscid species, specifically *G. mendeli* n. sp. and *G. steineri* n. sp. from *N. biguttatus*, in addition to *G. prikrylovae* n. sp. and *G. scholzi* n. sp. from *P. promelas.* Two other *Gyrodactylus* spp. were described, each from a single leuciscid species – specifically, *G. kuchtai* n. sp. from *C. neogaeus* and *G. lummei* n. sp. from *C. spadiceum.* Finally, two other species were described, each found on two leuciscid species – specifically, *G. hanseni* n. sp. from *L. chrysocephalus* and *S. atromaculatus*, and *G. huyseae* n. sp. from *L. chrysocephalus* and *N. hudsonius*. The remaining eight potentially new species were morphologically and genetically (when DNA sequences were available) characterized to provide background for further investigations when additional samples are obtained. Presently, insufficient sample sizes with respect to these parasites preclude accurate species descriptions. They concern two undescribed species found to parasitize *C. spadiceum*, a single species from each of *C. neogaeus*, *C. venusta*, the Mississippi silvery minnow *Hybognathus nuchalis* Agassiz, 1855 and *Lythrurus* sp., and finally three undescribed species hosted by *R. atratulus* (see below).

Combining morphological and genetic analyses has nowadays become a common practice in monogenean species identification [5]. While both analyses individually show specific limitations, together they provide more accurate taxonomic support for *Gyrodactylus* spp. [55]. In this group, the morphological features used for species description are related almost exclusively to the shape, size, and proportions of several haptoral structures [59]. Considering the high species richness of *Gyrodactylus* [2], non-significant morphological variations are expected, these causing insoluble confusions in species distinction [26]. DNA segments such as the ITS regions and, to a lesser degree, the 18S rDNA have been shown to be successful markers for revealing new species, and for assessing intraspecific variability [18, 76, 110].

In accordance with Ziętara et al. [110], we confirmed the utility of each part of the ITS regions in *Gyrodactylus* spp. delimitation. It should be noted that, in this study, some *Gyrodactylus* specimens were not subjected to both morphological and genetic analyses because of the limited sample size or unsuccessful sequencing. Moreover, morphological differences in the attachment organ are essential components of accurate species delimitation in *Gyrodactylus* [109]. Controversial taxonomy is mainly related to high levels of morphological intraspecific variation and interspecific similarities. Ziętara and Lumme [109] suggested that species delimitation is closely related to host specificity. In our study, *G. hanseni* n. sp. was found on *L. chrysocephalus* and *S. atromaculatus*, both inhabiting Midwestern localities. The weak sample size of *G. hanseni* n. sp. specimens parasitizing the latter host could suggest an accidental infection during the manipulation of the fish. However, a disparity was noticed in the haptoral morphology of *G. hanseni* n. sp. specimens in two host species, while although weak, a genetic variation was recovered. This may be connected with the fact that variation in haptoral sclerites may facilitate the colonization of new host species without corresponding genetic diversification, which is partly in accordance with a study on monogenean communities parasitizing marine Sparidae [44]. The presence of *G. hanseni* n. sp. on unrelated fish hosts, but yet occurring in overlapping habitats, makes it a generalist species that was probably host-switched to additional host species from the main host species [95]. Larger sample sizes of this *Gyrodactylus* species, covering a wider distributional range of the hosts, and subsequent host-parasite cophylogenetic analyses potentially feasible in the future will help in revealing the scenario of *Gyrodactylus* diversification.

Results obtained for *G. huyseae* n. sp. from *L. chrysocephalus* and *N. hudsonius* were ambiguous. Indeed, specimens of *G. huyseae* n. sp. overlap in each of their host geographical range [77], haptoral morphology (no consistent shape/size variation), and preferences for the site of infection (infect the fins in both cases). Contrariwise, genetic data obtained for *G. huyseae* n. sp. are questionable with respect to the variation in the sequences of each of ITS regions where the genetic variation slightly exceeded the limit value, and in that of the 18S rDNA where a single mutation was present. Genetic distances higher than 1% could indicate interspecific differentiation when these differences are accompanied by a meaningful ecological pattern [108]. For distinct systems, different limiting values for intra- and interspecific variation based on the ITS sequences were observed; i.e., 1.14% for African *Gyrodactylus* spp. [89], while up to 5% was retained for Neotropical communities [96]. Considering our results for *G. huyseae* n. sp., two scenarios are possible. The first one is that the specimens parasitizing *L. chrysocephalus* and *N. hudsonius* represent two distinct species. The weak sample size did not allow us to safely discriminate specimens of two potential species and elaborate two distinct formal descriptions. Nevertheless, in this case the mismatches defined on morphology and on molecular genetics may reflect a complex speciation process of diversification involving a recent/ongoing gene flow. The second scenario is that *G. huyseae* n. sp. is a cryptic generalist species parasitizing unrelated but geographically overlapping fish hosts. The shared evolutionary history of the two leuciscid representatives may have played a role in sharing the same *Gyrodactylus* spp. Indeed, *Luxilus* has long been considered a subgenus of *Notropis* until Mayden [62] elevated the former to genus level. Moreover, hybridization may easily occur between *L. chrysocephalus* and *N. hudsonius* due to overlapping habitats for spawning [72]. Revision of the taxonomic status of *G. huyseae* n. sp. using a higher sample size is suggested. Further, the use of mitochondrial markers like COI and microsatellites would detect potential introgression between parasite populations.

Our results revealed the complete conservation of 18S rDNA sequences among pairwise *G. colemanensis*; *G. hanseni* n. sp.; the unidentified *Gyrodactylus* sp. 1 “*R. atratulus*” and *Gyrodactylus* sp. 2 “*R. atratulus*”; and the previously published *Gyrodactylus* sp. (KT149284), all so far found to parasitize distinct cypriniform fish families (see above). This result was supported by haptoral morphology, but we revealed variations in the ITS sequences. Gilmore et al. [31] reported high similarity in 18S rDNA sequences; however, they considered this gene useful for the taxonomy and phylogeny of Nearctic *Gyrodactylus* spp. Our results based on partial sequences of this gene demonstrate that genetic convergence considerably reduces species-level resolution. We showed that some published 18S rDNA sequences of *Gyrodactylus* spp. fully matched the newly generated sequences representing morphologically distinguishable species, and that ITS regions are an accurate tool for species delimitation.

In two distinct areas of the Nearctic region, each of two populations of *P. promelas* harbored one of the two newly described *Gyrodactylus* specimens, *G. prikrylovae* n. sp. or *G. scholzi* n. sp. As already observed for several species (see above), genetic variation based on their ITS sequences was around the limiting value for species delimitation, whilst their 18S sequences were identical. *Gyrodactylus prikrylovae* n. sp. and *G. scholzi* n. sp. were confidently separated according to differential morphological features related to the shapes of the dorsal and ventral bars and ITS sequences where 1.6–1.7% (15 bp out of 1016 bp) of inter-species genetic variation was found. Moreover, the nBLAST search using ITS sequences (although with weak coverage) showed that *Gyrodactylus* sp. (AY099507) from Northwestern *P. promelas* [31] was genetically closer to *G. prikrylovae* n. sp. than to *G. scholzi* n. sp. However, at this stage, it remains difficult to accurately assign *Gyrodactylus* sp. to one of these newly described species.

Our study revealed some morphological features typical for Nearctic *Gyrodactylus* lineages. This was the case with the knob observed on the ventral bar of *G. scholzi* n. sp., *G. lummei* n. sp., and *G. stunkardi*, a feature already reported together with a few other haptoral traits in *Gyrodactylus* spp. in this region [55]. Investigating the phylogenetic relationships among *Gyrodactylus* spp. from different geographical regions and mapping their haptoral morphology onto phylogeny would reveal whether such specific characters potentially delimit some of the Nearctic lineages. Interestingly, two species parasitizing *C. commersonii* from closely North-eastern streams, namely *G. ellae* n. sp. and *G. hamdii* n. sp., showed distinct morphotypes. In addition, morphometric data regarding anchors, the ventral bar, and marginal hooks indicated sufficient evidence to support the identification of *G. hamdii* n. sp. and *G. commersoni* [99], both from distant *C. commersonii* populations, as two valid species. When considering ventral bar features like the absence of lateral processes and the spine-like shape of the membrane in *G. ellae* n. sp., which resembles the Palearctic *G. elegans* haptoral group [59], *G. ellae* n. sp. and *G. hamdii* n. sp. are highly distinguishable on the basis of morphology, which is in accordance with the variability in their ITS and 18S rDNA sequences. Furthermore, the haptoral morphology exhibited by *G. ellae* n. sp. highly resembles that of *G. kuchtai* n. sp. and the undescribed *Gyrodactylus* sp. “*C. neogaeus*”, both parasitizing phylogenetically distant cypriniform hosts. Their morphological similarity was in line with the pattern observed for DNA sequences, which showed relatively weak variation. These observations imply that *Gyrodactylus* spp. parasitizing distinct hosts are more closely related to each other than species occurring on the same host, which was the case of Eurasian *Gyrodactylus* of gobies [42]. For other morphological traits, the marginal hooks exhibited by *G. kuchtai* n. sp. and *Gyrodactylus* sp. from *C. neogaeus* seem to be very similar to those of many representatives from *Phoxinus* spp. [2, 83], which may mirror phylogenetical and historical relationships between European and Nearctic minnows. An exhaustive molecular study involving a larger sample size of *Gyrodactylus* spp., supplemented by the mapping of haptoral morphology onto parasite phylogeny, would contribute to the tracking of the morphological evolution and phylogenetic origin of Nearctic *Gyrodactylus*, its diversification on the continental scale, and its ancient biogeographical contacts with Eurasian congeners.

Finally, new data are presented in this study of the Nearctic *Gyrodactylus* fauna. Due to a lack of sufficient mounted specimens representing some species that may be potentially new to science, we present a brief morphological characterization, supplemented by genetic information (when available), for eight species without any formal description. Concerning previous records of *Gyrodactylus* spp. in the Nearctic region, we document seven species and present their haptoral morphology supplemented by genetic characterization. We report the presence of *G. atratuli*, *G. stunkardi,* and *G. dechtiari*, all previously known from leuciscid hosts with wide distributional ranges in the Nearctic area [22, 23, 32], and *G. spathulatus* and *G. wardi* mostly on widely-dispersed *Catostomus* spp. [20–25, 39, 49, 66, 71, 99]. For *G. atratuli* collected from two distant localities, we revealed morphological intraspecific variation likely related to isolation-by-distance. As already suggested above, the presence of *Gyrodactylus* spp. on non-congeneric hosts could be the result of host switching. This scenario remains valid for the generalist *G. stunkardi* found to parasitize *R. atratulis* in this study, and previously identified mainly on congeneric [22, 32], and rarely on geographically and phylogenetically distant catostomid [49], as well as on perciform fish hosts [23]. It should be noted that *C. occidentalis* is restricted to Californian freshwater systems, which could identify *G. stunkardi* as an alien parasite in the western USA. Another *Gyrodactylus* spp. documented in our study was *G. colemanensis*. Morphology and genetics supported its presence in a Northeastern *E. maxillingua* population. Surprisingly, this parasite species was so far restricted to Nearctic captive salmonid hosts, which makes our findings involving representatives of wild leuciscid fish unique. This could be the consequence of ecological host switching followed by fish translocation for farming purposes, since the geographical distribution of salmonids overlaps with that of *E. maxillingua* [77]. As suggested by Leis et al. [54], the newly generated ITS sequences confirmed the identity of *Gyrodactylus* sp. (KT149288) as *G. variabilis*, a species previously recorded from captive *N. crysoleucas* and originally described on the same host introduced to California. This study also allowed us to identify this species on *N. crysoleucas* across a wide native distributional range (USA and Canada). As obtained for *G. hanseni* n. sp., *G. variabilis* in our study showed variations in the ITS sequences around the limiting value for species delimitation. The host range recorded so far for *G. hanseni* n. sp. and *G. variabilis* and their morphological features, however, support species status for these species.

In conclusion, our study demonstrates the limited knowledge currently available on the diversity of monogenean parasites in wild-living freshwater fish in the Nearctic region. Using an integrative approach combining the morphological investigation of taxonomically relevant traits with conventional molecular markers suitable for *Gyrodactylus* spp. delineation, our contribution identified 10 new species and a further eight formally undescribed species. This reveals the hidden diversity of *Gyrodactylus* parasites in Nearctic cypriniform fish. We further highlight the importance of future studies in the Nearctic region to investigate the diversity of monogenean parasites and their phylogenetic relatedness, host specificity, and morphological evolution.

## Figures and Tables

**Figure 6:**
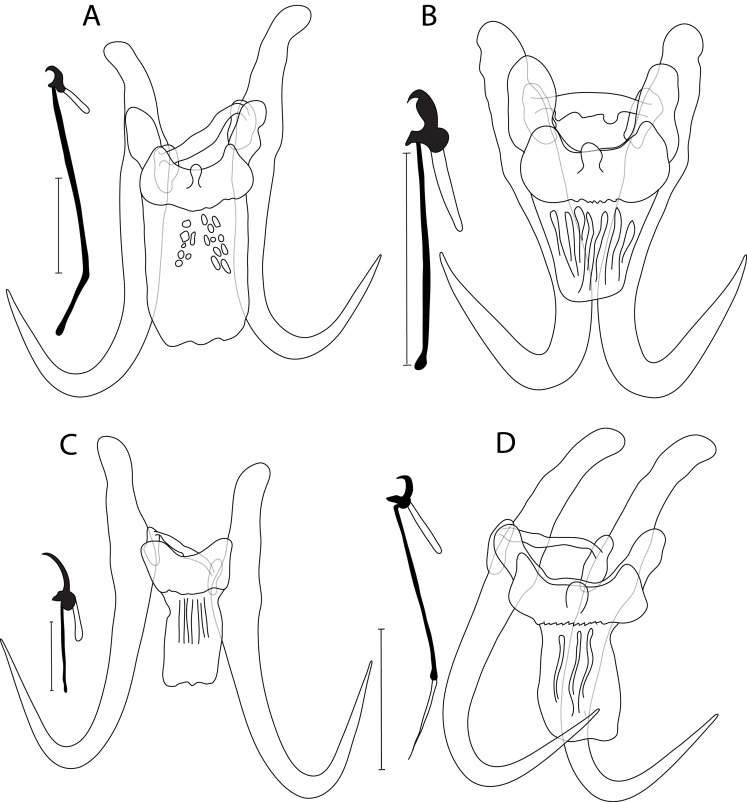
Drawing of haptoral sclerotized structures of undescribed *Gyrodactylus* spp.: (A) *Gyrodactylus* sp. 1 “*C. spadiceum*”; (B) *Gyrodactylus* sp. 2 “*C. spadiceum*”; (C) *Gyrodactylus* sp. “*C. neogaeus*”; and (D) *Gyrodactylus* sp. “*C. venusta*”. Scale bar = 20 μm.

**Figure 7:**
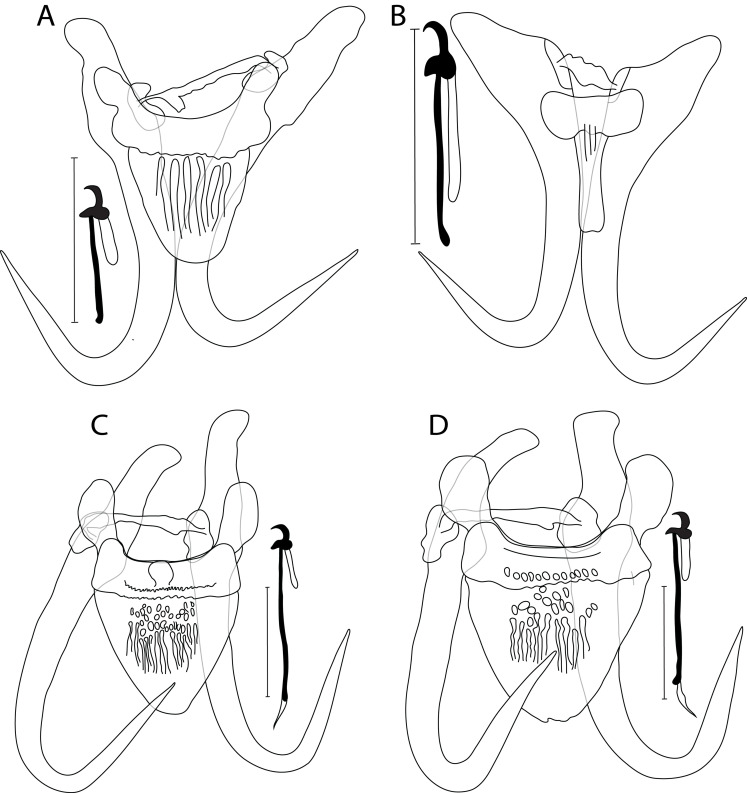
Drawing of haptoral sclerotized structures of undescribed *Gyrodactylus* spp.: (A) *Gyrodactylus* sp. “*H. nuchalis*”; (B) *Gyrodactylus* sp. “*Lythrurus* sp.”; (C) *Gyrodactylus* sp. 1 “*R. atratulus*”; and (D) *Gyrodactylus* sp. 2 “*R. atratulus*”. Scale bar = 20 μm.

## Data Availability

The data supporting the conclusions of this article are included within the article. The type-material and vouchers of species investigated in this study were deposited in the Muséum National d’Histoire Naturelle (MNHN, Paris, France) (see taxonomic summaries for details on repositories and accession numbers). DNA sequence data are available in the GenBank Nucleotide Database under accession numbers listed in each description section of *Gyrodactylus* spp. (see above).

## Supplementary material

The supplementary material of this article is available at https://www.parasite-journal.org/10.1051/parasite/2022016/olm. *Table S1.* Matrix of pairwise genetic distances (*p*-distance in 1st line) based on alignment of the ITS regions (848 bp) and number of variable nucleotides (2nd line), including Gyrodactylus spp. investigated in this study (in bold) and their closest hits revealed by nBLAST search, listed in Table 2 (below diagonal) and based on newly generated sequences (1016 bp long) of Gyrodactylus spp. (above diagonal). ^H1^ = L. chrysocephalus; ^H2^ = S. atromaculatus; ^H3^ = N. hudsonius; ^L1^ = Morrys Creek (Wisconsin); ^L2^ = Oaks Creek (New York); ^L3^ = Mink River (Wisconsin) (see Table 1 for fish sampling details). *Table S2*. Matrix of pairwise genetic distances (*p*-distance) based on the alignment of the 18S rDNA nucleotide sequences (435 bp long) (below diagonal) and number of variable nucleotides (above diagonal), including *Gyrodactylus* spp. investigated in this study (in bold) and their closest hits revealed by nBLAST search and listed in Table 2. ^H1^ = *L. chrysocephalus*; ^H2^ = *S. atromaculatus*; ^H3^ = *N. hudsonius* (see Table 1 for fish sampling details).

## Appendix

### Undescribed Nearctic *Gyrodactylus* spp.

For the description of new species, at least three specimens are formally required. Due to the small number of *Gyrodactylus* specimens and the poor quality of fixed specimens on mounted slides, eight potentially new species from Nearctic (USA) cypriniform hosts (Table 1) were mostly characterized (though not formally described) on the basis of haptoral sclerite morphometry and shape morphology. All *Gyrodactylus* specimens were fixed in GAP (see Material and methods). Parasitological parameters (prevalence and intensity of infection) were not calculated. Differential diagnosis is presented, supplemented by 18S and ITS sequences when available.

#### *Gyrodactylus* sp. 1 “*C. spadiceum*” (Fig. 6A)

Host: *C. spadiceum* (Leuciscidae)

Site of infection: gills

Locality: Caddo River, Polk County, Arkansas, USA

Host GenBank accession number: OR270016

Parasite GenBank accession number: 18S rDNA: OR269973; ITS: OR270016

##### Morphological characterization

Haptor subcircular, anchor base may possess folds, tips slightly curved outward, total length 75.4 (*n* = 1); shaft slightly bowed, length 56.9 (*n* = 1); point curved and elongate, length 30.2 (*n* = 1); root relatively long, length 27.5 (*n* = 1). Ventral bar with blunt lateral processes extending out of bar, total length 25.2 (*n* = 1), median part with a knob, width 9.2 (*n* = 1); distance between tips 32.4 (*n* = 1); membrane (shield) rectangular, plate-like extending posteriorly almost length of anchor shaft, tapering slightly posteriorly with lateral margins and rows of ovate to rectangular ridges ending almost halfway, length 28.9 (*n* = 1). Dorsal bar variably bent, attenuated ends inserted into terminal plates, total length 23.6 (*n* = 1). Marginal hooks total length 59.6 (*n* = 1); sickle foot poorly-developed with globose and dipped down heel, relatively inconspicuous blunt toe and shelf; sickle proper over two times the thickness of toe base, shaft length 3.6 (*n* = 1); sickle length to shaft attachment 5.6 (*n* = 1); sickle proximal width 4.7 (*n* = 1); sickle distal width 2.6 (*n* = 1); point relatively thick, short and weekly curved, length 2.3 (*n* = 1); filament loop short, extending about 1/7 handle length, length 7.8 (*n* = 1); handle relatively long, deflected near proximal end, length 54.6 (*n* = 1). MCO not observed.

##### Differential diagnosis

Morphological features indicating a potentially new species parasitizing *C. spadiceum*, referred to herein as *Gyrodactylus* sp. 1 “*C. spadiceum*” (Fig. 6A) and its newly described congener *G. lummei* n. sp., closely related but still separated at species level, were detailed in the species description of *G. lummei n. sp*. Although the haptoral sclerites are of similar shape in *Gyrodactylus* sp. 1 “*C. spadiceum*” and *G. campostomae*, both known from *Campostoma* spp. [10, 49, 102], the former species mainly differs by its larger anchors (75.4 μm in *Gyrodactylus* sp. 1 “*C. spadiceum”* vs. 45 μm in *G. campostomae*).

Fragments covering ITS1 (371 bp), 5.8S (157 bp), ITS2 (390 bp), and 18S rDNA (439 bp) were successfully sequenced for a single specimen of *Gyrodactylus* sp. 1 “*C. spadiceum*” parasitizing South-central *C. spadiceum* (Arkansas, USA) (Table 1). No close hit was recovered when searching on nBLAST using sequences of the ITS and 18S rDNA.

#### *Gyrodactylus* sp. 2 “*C. spadiceum*” (Fig. 6B)

Host: *C. spadiceum* (Leuciscidae)

Site of infection: fins

Locality: Butcherknife Creek and Caddo River, both in Polk County, Arkansas, USA

Host GenBank accession number: OR270017

Parasite GenBank accession number: 18S rDNA: OR269974; ITS: OR270017

##### Morphological characterization

Haptor subcircular, anchor base may possess folds, total length 33.4 (32–35.1; *n* = 2); shaft relatively thick, slightly bowed, length 25 (24–26.6; *n* = 2); point curved and elongate, length 15.1 (15–15.2; *n* = 2); root short, length 8.3 (6.9–10; *n* = 2). Ventral bar with blunt lateral processes extending out of bar, length 13.5 (13.3–13.7; *n* = 2), median part with a knob, width 4.8 (4.3–5.3; *n* = 2); distance between tips 17.2 (16.3–18.2; *n* = 2); membrane (shield) trapezoid, extending posteriorly almost 1/2 length of anchor shaft, tapering posteriorly, length 12.3 (10.1–14.6; *n* = 2). Dorsal bar relatively thick variably bent, with projections near each end, attenuated ends inserted into terminal plates, total length 13.7 (13.3–14.1; *n* = 2). Marginal hooks total length 23.3 (22.7–23.8; *n* = 2); sickle significant but disproportionate with well-developed and globose heel, triangular and pointed toe, prominent shelf; sickle proper thick, swollen with constriction near point insertion, shaft length 3.6 (3.5–3.8; *n* = 2); sickle length to shaft attachment 4.8 (4.7–4.9; *n* = 2); sickle proximal width 3.9 (3.8–3.9; *n* = 2); sickle distal width 2.9 (2.8–2.9; *n* = 2); point reduced and thick, length 1.3 (1.2–1.4; *n* = 2); filament loop extending about 2/3 of handle length, length 7.6 (7.2–8; *n* = 2); handle length 18.4 (17.3–18.9; *n* = 2). MCO not observed.

##### Differential diagnosis

*Gyrodactylus* sp. 2 “*C. spadiceum*” (Fig. 6B) and its previously listed congener *Gyrodactylus* sp. 1 “*C. spadiceum*” (Fig. 6A) exhibit highly distinguishable shapes of the haptoral structures from each other, especially that of the ventral bar and marginal hooks; these differences were already detailed when *Gyrodactylus* sp. 2 “*C. spadiceum*” was compared to *G. campostomae* (see above).

Fragments covering ITS1 (366 bp), 5.8S (157 bp), ITS2 (399 bp), and 18S rDNA (439 bp) were successfully sequenced for a single specimen of *Gyrodactylus* sp. 2 “*C. spadiceum*” parasitizing South-central *C. spadiceum* (Arkansas, USA) (Table 1). No close hit was recovered using nBLAST search (Table 2), and considerable genetic variation was obtained in sequences of the ITS and 18S rDNA of the representatives of *Gyrodactylus* sp. 2 “*C. spadiceum*” and all remaining species (Tables S1 and S2).

#### *Gyrodactylus* sp. “*C. neogaeus*” (Fig. 6C)

Host: *C. neogaeus* (Leuciscidae)

Site of infection: fins

Locality: Mink River, Door County, Wisconsin, USA

Host GenBank accession number: OR270018

Parasite GenBank accession number: 18S rDNA: OR269975; ITS: OR270018

##### Morphological characterization

Haptor subcircular, anchor base seems lacking folds, total length 67.7 (*n* = 1); shaft slightly bowed, length 51.9 (*n* = 1); point curved and elongate, length 28.5 (*n* = 1); root moderately long, length 22.7 (*n* = 1). Ventral bar lacking lateral processes, total length 18.6 (*n* = 1), median width 7.1 (*n* = 1); membrane (shield) almost rectangular, constricted near insertion, extending proximally about 1/2 length of anchor shaft with fine longitudinal striations, length 12.9 (*n* = 1). Dorsal bar variably bent, slightly constricted at midpoint with attenuated ends inserted into terminal plates, total length 14.5 (*n* = 1). Marginal hooks total length 33 (*n* = 1); sickle foot disproportionate with globose and dipped down heel, straight and triangular toe, conspicuous shelf; sickle proper 1/2 the thickness of toe base, shaft length 4.5 (*n* = 1); sickle length to shaft attachment 6.3 (*n* = 1); sickle proximal width 4.2 (*n* = 1); sickle distal width 4.6 (*n* = 1); far reaching, short and weekly curved point, length 1.2 (*n* = 1); filament extending about 1/2 handle length, length 9.4 (*n* = 1); handle length 13.7 (*n* = 1). MCO not observed.

##### Differential diagnosis

In addition to *G. kuchtai* n. sp. described in this study, *C. neogaeus* was shown to host an additional species, which is undescribed for lack of sufficient material. The morphological features making *Gyrodactylus* sp. “*C. neogaeus*” (Fig. 6C) different from *G. ellae* n. sp. were already detailed in the *G. ellae* n. sp. description. Previously, it was found that *C. neogaeus* occurring in Missouri watersheds hosted *G. nebraskensis* Mayes, 1977, the sole representative known from this host so far [65]. *Gyrodactylus* sp. “*C. neogaeus*” is distinguishable from *G. nebraskensis* in having (i) larger anchors (67.7 μm in *Gyrodactylus* sp. “*C. neogaeus*” vs. 44–48 μm in *G. nebraskensis*), (ii) dorsal bar lacking projections, and (iii) longer hooks of obviously different shape (33 μm in *Gyrodactylus* sp. “*C. neogaeus*” vs. 19–21 μm in *G. nebraskensis*).

Fragments covering ITS1 (355 bp), 5.8S (157 bp), ITS2 (350 bp), and 18S rDNA (449 bp) were successfully sequenced for a single specimen of *Gyrodactylus* sp. “*C. neogaeus*” parasitizing *C. neogaeus* from a Midwestern location (Wisconsin, USA) (Table 1). nBLAST search did not indicate any close hit to *Gyrodactylus* sp. “*C. neogaeus*” (Table 2), while the newly described *G. ellae n. sp.* from *C. commersonii* was found to be its closest match based on the 18S rDNA sequences (*p*-distances = 0.9%, 4 bp; Table S2).

#### *Gyrodactylus* sp. “*C. venusta*” (Fig. 6D)

Host: *C. venusta* (Leuciscidae)

Site of infection: fins

Locality: Pascagoula River, Pascagoula, Mississippi, USA

##### Morphological characterization

Haptor subcircular, anchors not in natural position, base may possess folds, tips slightly curved outward, total length 57.9 (*n* = 1); shaft slightly bowed, length 33.9 (*n* = 1); point curved and elongate, length 21.7 (*n* = 1); root long, length 23.3 (*n* = 1). Ventral bar with blunt lateral processes extending out of bar, total length 20.1 (*n* = 1), median part with a knob, width 6.1 (*n* = 1); distance between tips 26.3 (*n* = 1); membrane (shield) rectangular, slightly constricted near insertion, extending posteriorly about 1/2 length of anchor shaft, slightly rounded posteriorly with fine longitudinal striations, length 18.3 (*n* = 1). Dorsal bar variably bent, attenuated ends inserted into terminal plates, total length 17.8 (*n* = 1). Marginal hooks total length 33 (*n* = 1); sickle foot relatively poorly developed with weekly globose heel, prominent toe and conspicuous shelf; sickle proper almost as thick as toe base, shaft length 3.6 (*n* = 1); sickle length to shaft attachment 4.6 (*n* = 1); sickle proximal width 2.7 (*n* = 1); sickle distal width 3.2 (*n* = 1); point weekly thick and curved, length 1.1 (*n* = 1); filament loop extending about 1/3 of handle length, length 10 (*n* = 1); handle ending with a filament in its posterior part, length 28.6 (*n* = 1). MCO not observed.

##### Differential diagnosis

*Gyrodactylus baeacanthus* Wellborn & Rogers, 1967 was, so far, the sole known species to parasitize *C. venusta* [103]. *Gyrodactylus* sp. “*C. venusta*” (Fig. 6D) and *G. baeacanthus* were shown to occur in connected Southern (Mississippi and Alabama, USA) freshwater habitats ([21] and this study). The main morphological features that discriminate *Gyrodactylus* sp. “*C. venusta*” from *G. baeacanthus* are (i) larger anchors (57.9 μm in *Gyrodactylus* sp. “*C. venusta*” vs. 27–32 μm in *G. baeacanthus*), (ii) a ventral bar with well-developed processes, unlike that of *G. baeacanthus*, (iii) a longer ventral bar membrane with a knob (18.3 μm in *Gyrodactylus* sp. “*C. venusta*” vs. 7–9 μm without a knob in *G. baeacanthus*), and (iv) a marginal hook filament in the handle proximal part in *Gyrodactylus* sp. “*C. venusta*”, a feature not observed in *G. baeacanthus*). Efforts to generate the 18S and ITS sequences for *Gyrodactylus* sp. “*C. venusta*” were unsuccessful.

#### *Gyrodactylus* sp. “*H. nuchalis*” (Fig. 7A)

Host: *H. nuchalis* (Leuciscidae)

Site of infection: gills

Locality: Pascagoula River, Pascagoula, Mississippi, USA

Host GenBank accession number: OR270019

Parasite GenBank accession number: 18S rDNA: OR269976; ITS: OR270019

##### Morphological characterization

Haptor subcircular, anchor base may possess folds, total length 43.9 (*n* = 1); shaft relatively bowed, length 33.5 (*n* = 1); point curved and elongate, length 19.3 (*n* = 1); root moderately short, length 11.8 (*n* = 1). Ventral bar with blunt lateral processes extending out of bar, total length 17.8 (*n* = 1), median width 4.3 (*n* = 1); distance between tips 21.1 (*n* = 1); membrane (shield) semioval, extending posteriorly almost 1/2 length of anchor shaft, tapering posteriorly for rounded edges, length 13.2 (*n* = 1). Dorsal bar relatively straight, with projections near each end, attenuated ends inserted into terminal plates, total length 18.4 (*n* = 1). Marginal hooks total length 22.7 (*n* = 1); sickle foot significant with straight globose heel, prolonged triangular toe, inconspicuous shelf; sickle proper of 1/2 the thickness of toe base, shaft length 2.9 (*n* = 1); sickle length to shaft attachment 4.2 (*n* = 1); sickle proximal width 3.1 (*n* = 1); sickle distal width 2.5 (*n* = 1); point relatively short and weekly curved, length 1.4 (*n* = 1); filament loop extending about 1/2 handle length, length 6.5 (*n* = 1); handle length 18.5 (*n* = 1). MCO not observed.

##### Differential diagnosis

This study presents ectoparasitic monogeneans from *H. nuchalis* for the first time. Morphologically, *Gyrodactylus* sp. “*H. nuchalis*” (Fig. 7A) looks similar to *G. scholzi* n. sp. parasitizing *P. promelas*, but *Gyrodactylus* sp. “*H. nuchalis*” is distinguishable by the absence of a rounded knob in the median part of the ventral bar (present in *G. scholzi* n. sp.).

Fragments covering ITS1 (377 bp), 5.8S (157 bp), ITS2 (391 bp), and 18S rDNA (439 bp) were successfully sequenced for a single specimen of *Gyrodactylus* sp. “*H. nuchalis*” parasitizing South-eastern *H. nuchalis* (Mississippi, USA) (Table 1). No close congener to *Gyrodactylus* sp. “*H. nuchalis*” was revealed using nBLAST and *p-*distances based on sequences of the ITS and 18S rDNA (Tables 2, S1, S2).

#### *Gyrodactylus* sp. “*Lythrurus* sp.” (Fig. 7B)

Host: *Lythrurus* sp. (Leuciscidae)

Site of infection: gills

Locality: Roark Creek, Polk County, Arkansas, USA

##### Morphological characterization

Haptor subcircular, anchor base lacking folds, total length 32.9 (32.1–34.3; *n* = 3); shaft relatively straight, length 26 (25–27; *n* = 3); point curved, elongate, length 26 (25–27; *n* = 3); root short, length 13.5 (12.9–13.9; *n* = 3). Ventral bar lacking lateral processes, total length 10.6 (13.2–10.8; *n* = 2), median width 4 (3.9–4.1; *n* = 2); membrane (shield) narrow, lingulate-like, extending proximally about 1/2 length of anchor shaft, length 12.6 (12.4–12.6; *n* = 2). Dorsal bar relatively short and thick, variably bent, slightly constricted at midpoint with attenuated ends inserted into terminal plates, total length 10.4 (8.9–12.9; *n* = 3). Marginal hooks total length 20.9 (20.7–21.1; *n* = 3); sickle relatively significant with moderately-developed globose heel, triangular and curved toe, conspicuous shelf; sickle proper almost three times the thickness of toe base, length 4.1 (3.7–4.7; *n* = 3); sickle length to shaft attachment 4.9 (3.6–5.7; *n* = 3); sickle proximal width 3.5 (3.3–3.8; *n* = 3); sickle distal width 2.9 (2.6–3; *n* = 3); point relatively short and well-curved, length 1.8 (1.6–2; *n* = 3); filament loop long, extending almost whole handle length, length 11 (9–12.7; *n* = 3); handle length 15.5 (15.2–16.1; *n* = 3). MCO not observed.

##### Differential diagnosis

The species (Fig. 7B) could not be formally described mostly due to the poor quality of the slide-mounted specimens. *Gyrodactylus lythruri* Rogers, 1975 is the sole species known from the Nearctic pretty shiner *L. bellus* (Hay, 1881) (type-host) and blacktip shiner *L. atrapiculus* (Snelson, 1972) inhabiting Alabama rivers [86]. The undescribed species is clearly distinguishable from *G. lythruri*, especially by its differently shaped haptoral sclerites. The haptoral morphology shown by *Gyrodactylus* sp. “*Lythrurus* sp.” remains largely that of *G. lingulatus* Rogers, 1968 described from the Alabama hog sucker *Hypentelium etowanum* (Jordan, 1877) [87], but the former undescribed species is mainly distinguishable by its shorter anchors (32.1–34.3 μm in *Gyrodactylus* sp. “*Lythrurus* sp.” vs. 61–68 μm in *G. lingulatus*). Efforts to generate the 18S and ITS sequences for *Gyrodactylus* sp. “*Lythrurus* sp.” were unsuccessful.

#### *Gyrodactylus* sp. 1 “*R. atratulus*” (Fig. 7C)

Host: *R. atratulus* (Leuciscidae)

Site of infection: fins

Locality: Leatherstocking Creek, Otsego, New York state, USA

Host GenBank accession number: OR270020

Parasite GenBank accession number: 18S rDNA: OR269977; ITS: OR270020

##### Morphological characterization

Haptor subcircular, anchors not in natural position, base may possess folds, total length 66.5 (67.1–69.9; *n* = 2); shaft slightly bowed, length 48.8 (46.9–50.6; *n* = 2); point curved and elongate, length 31.8 (31.6–32.1; *n* = 2); root moderately long, length 21.7 (20.7–22.7; *n* = 2). Ventral bar with blunt lateral processes extending out of bar, length 27.9 (27.8–27.9; *n* = 2), median part with visible knob, width 7.8 (7.7–7.8; *n* = 2); distance between tips 32.8 (32.5–33.1; *n* = 2); membrane (shield) almost triangular, extending posteriorly almost 1/2 length of anchor shaft, tapering posteriorly with rows of ovate to rectangular ridges, length 23 (22.7–23.3; *n* = 2). Dorsal bar variably bent, constricted at almost midpoint with projections almost near each ending, attenuated ends inserted into terminal plates, total length 26.7 (24.6–28.8; *n* = 2). Marginal hooks total length 31.5 (31.3–31.6; *n* = 2); sickle foot significant with globose heel, prominent triangular toe, conspicuous shelf; sickle proper almost as thick as toe base, shaft length 3.6 (3.5–3.8; *n* = 2); sickle length to shaft attachment 4.7 (4.6–4.7; *n* = 2); sickle proximal width 3.8 (3.6–4; *n* = 2); sickle distal width 3.1 (3–3.2; *n* = 2); point thin and weekly-curved, length 2 (1.9–2.2; *n* = 2); filament loop extending about 2/3 of handle length, length 7.6 (7.2–8; *n* = 2); handle ending with a filament in its posterior part, length 26.7 (26.5–26.8; *n* = 2). MCO with 5–6 spinelets.

##### Differential diagnosis

The combination of haptoral morphology and genetics shows *Gyrodactylus* sp. 1 “*R. atratulus*” (Fig. 7C) to be a potentially new species awaiting formal description. This species together with *G. atratuli* share *R. atratulus* as host [84]. In terms of morphology, *Gyrodactylus* sp. 1 “*R. atratulus*” is differentiated by the presence of a knob in the ventral bar, a feature lacking in *G. atratuli*. Similarly, *Gyrodactylus* sp. 1 “*R. atratulus*” differs from *G. avalonia* Hanek & Threlfall, 1969 and G. *dechtiari* Hanek & Fernando, 1971, both reported from *R. atratulus* [32, 33], and on a range of Nearctic [22] and non-endemic European hosts [52], by its longer anchors (66.5–70.2 μm in *Gyrodactylus* sp. 1 “*R. atratulus*” vs. 40–43 μm in *G. avalonia* in [22] and [52]; 45 μm in G. *dechtiari*).

Fragments covering ITS1 (370 bp), 5.8S (157 bp), ITS2 (388 bp), and 18S rDNA (439 bp) were successfully sequenced for two specimens of *Gyrodactylus* sp. 1 “*R. atratulus*” parasitizing Northeastern *R. atratulus* (New York, USA) (Table 1). Newly-generated sequences were identical for each of the ITS regions and 18S rDNA. nBLAST search did not reveal any hit when considering the ITS region sequences, whereas those of 18S rDNA from both *G. colemanensis* (JF836090) [31] and *Gyrodactylus* sp. (KT149284) (identified as *G. huyseae* n. sp. in this study) parasitizing captive *N. crysoleucas* [54] were nearly identical to that of the undescribed specimen assigned to *Gyrodactylus* sp. 1 “*R. atratulus*” (Tables 2 and S2). *Gyrodactylus atratuli* also parasitizing *R. atratulus* was genetically the closest to *Gyrodactylus* sp. 1 “*R. atratulus*”, with a genetic variation in the ITS sequences above the limiting value [41, 108] (*p*-distances = 1.8%, 17 bp; Table S1). Sequences of the 18S rDNA from both undescribed species from *R. atratulus*, as well as from *G. hanseni* nov. sp. parasitizing *L. chrysocephalus* and *S. atromaculatus* were identical (Table S2).

#### *Gyrodactylus* sp. 2 “*R. atratulus*” (Fig. 7D)

Host: *R. atratulus* (Leuciscidae)

Site of infection: fins

Locality: Leatherstocking Creek, Otsego, New York, USA; Mink River, Door County, Wisconsin, USA

Host GenBank accession number: OR270021, OR270022

Parasite GenBank accession number: 18S rDNA: OR269978, OR269979; ITS: OR270021, OR270022

##### Morphological characterization

Haptor subcircular, anchors not in natural position, base may possess folds, total length 70.2 (*n* = 1); shaft slightly bowed, length 51.3 (*n* = 1); point curved and elongate, length 30.8 (*n* = 1); root moderately long, length 21.4 (*n* = 1). Ventral bar with blunt lateral processes extending out of bar, length 27.6 (*n* = 1), median width 8 (*n* = 1); distance between tips 46.1 (*n* = 1); membrane (shield) almost triangular, extending posteriorly almost 1/2 length of anchor shaft, tapering posteriorly with rows of ovate to rectangular ridges, length 37.8 (*n* = 1). Dorsal bar variably bent, constricted at almost midpoint with projections almost near each ending, attenuated ends inserted into terminal plates, total length 23.7 (*n* = 1). Marginal hooks total length 32.5 (*n* = 1); sickle foot significant but disproportionate with well-developed globose heel, triangular and slightly curved toe, prominent shelf; sickle proper as thick as toe base, shaft length 3.2 (*n* = 1); sickle length to shaft attachment 4.5 (*n* = 1); sickle proximal width 3.8 (*n* = 1); sickle distal width 3 (*n* = 1); point relatively thin and weekly curved, length 1.5 (*n* = 1); filament loop extending about 2/3 of handle length, length 7 (*n* = 1); handle ending with a filament in its posterior part, length 25.5 (*n* = 1). MCO not observed.

##### Differential diagnosis

Even though a total of three specimens were collected for *Gyrodactylus* sp. 2 “*R. atratulus*” (Fig. 7D), the morphology of two mounted specimens representing Midwestern sampling locality was unclear (poor-quality slides), which delayed the formal description of this species. Nevertheless, *Gyrodactylus* sp. 2 “*R. atratulus*” is highly similar to its congener *Gyrodactylus* sp. 1 “*R. atratulus*”, presented above, yet distinguishable by the lack of the knob in the median part of its ventral bar. Furthermore, the marginal hooks in both species appear to be of different shape, especially in the toe and shelf structures. This could not be thoroughly investigated due to the limited sample size. Compared to the species known from *R. atratulus*, *Gyrodactylus* sp. 2 “*R. atratulus*” is distinguishable by its longer anchors (66.5–70.2 μm in *Gyrodactylus* sp. 2 “*R. atratulus*” vs. 59–63 μm in *G. atratuli*; 40–43 μm in *G. avalonia* in [22, 52]; and 45 μm in G. *dechtiari*).

Fragments covering ITS1 (370 bp), 5.8S (157 bp), ITS2 (388 bp), and 18S rDNA (439 bp) were successfully sequenced for a single specimen of *Gyrodactylus* sp. 2 “*R. atratulus*” parasitizing Northeastern *R. atratulus* (New York, USA) and for two parasite specimens from Midwestern location (Wisconsin, USA) (Table 1). For each sampling locality, sequences of the ITS regions and 18S rDNA were identical. nBLAST search (Table 2) indicated similar hits found for *Gyrodactylus* sp. 1 “*R. atratulus*” (see above). On a geographical scale, weak intraspecific variation was found using the ITS sequences (Table S1), in contrast to 18S rDNA sequences which were identical (Table S2).
