# Supplementary material for: Revealing the hidden diversity of Gyrodactylus communities (Monogenea, Gyrodactylidae) from Nearctic Catostomidae and Leuciscidae fish hosts (Teleostei, Cypriniformes), with descriptions of ten new species
Source: Parasite. 2023 Sep 27;30:40. doi: 10.1051/parasite/2023035 (PMC10537664; doi:10.1051/parasite/2023035)
Supplement: Supplementary file 1 — Table S1. Matrix of pairwise genetic distances (p-distance in 1st line) based on alignment of the ITS regions (848 bp) and number of variable nucleotides (2nd line), including Gyrodactylus spp. investigated in this study (in bold) and their closest hits revealed by nBLAST search, listed in Table 2 (below diagonal) and based on newly generated sequences (1016 bp long) of Gyrodactylus spp. (above diagonal). H1 = L. chrysocephalus; H2 = S. atromaculatus; H3 = N. hudsonius; L1 = Morrys Creek (Wisconsin); L2 = Oaks Creek (New York); L3 = Mink River (Wisconsin) (see Table 1 for fish sampling details). [file parasite-30-40-s1.pdf]

Table S1 Matrix of pairwise genetic distances (*p*–distance in 1st line) based on alignment of ITS regions (848 bp) and number of variable nucleotides (2nd line), including *Gyrodactylus* species investigated in this study (**in bold**) and their closest hits revealed by nBLAST search, listed in Table 2 (below diagonal) and based on newly-generated sequences (1016 bp long) of *Gyrodactylus* species (above diagonal). H1 = *L. chrysocephalus;* H2 = *S. atromaculatus;* H3 = *N. hudsonius*; L1 =Morrys Creek (Wisconsin); L2 =Oaks Creek (New York); L3 =Mink River (Wisconsin) (see Table 1 for fish sampling details).

	(1)	(2)	(3)	(4)	(5)	(6)	(7)	(8)	(9)	(10)	(11)	(12)	(13)	(14)	(15)	(16)	(17)	(18)	(19)	(20)	(21)	(22)	(23)	(24)	(25)	(26)	(27)	(28)	(29)
**(1) *G. atratuli***		0.022<br>20		0.169<br>154	0.312<br>263	0.124<br>114	0.028<br>26	0.025<br>23	0.340<br>289	0.337<br>287	0.324<br>277	0.155<br>140	0.103<br>94	0.097<br>89	0.098<br>90	0.098<br>90	0.333<br>284	0.092<br>84	0.150<br>136	0.140<br>127	0.018<br>17	0.021<br>19	0.017<br>16	0.135<br>122	0.128<br>116			0.026<br>24	0.141<br>129
**(2) *G. colemanensis***	0.021<br>17			0.170<br>155	0.314<br>265	0.138<br>127	0.043<br>40	0.040<br>37	0.336<br>287	0.334<br>285	0.324<br>277	0.156<br>141	0.107<br>98	0.105<br>96	0.106<br>97	0.108<br>99	0.329<br>282	0.098<br>90	0.148<br>134	0.142<br>129	0.036<br>33	0.034<br>31	0.030<br>28	0.142<br>129	0.133<br>121			0.041<br>38	0.152<br>140
(3) *G. colemanensis*[1]	0.018<br>15	0.004<br>3																											
**(4) *G. dechtiari***	0.152<br>122	0.150<br>121	0.153<br>123		0.342<br>289	0.184<br>176	0.170<br>155	0.171<br>156	0.350<br>297	0.349<br>296	0.347<br>297	0.199<br>181	0.163<br>153	0.177<br>165	0.177<br>164	0.181<br>169	0.345<br>295	0.163<br>150	0.199<br>181	0.194<br>178	0.166<br>151	0.165<br>150	0.164<br>149	0.185<br>169	0.185<br>169			0.169<br>154	0.201<br>192
**(5) *G. ellae* sp. nov.**	0.293<br>221	0.289<br>219	0.292<br>221	0.311<br>234		0.322<br>273	0.314<br>265	0.316<br>266	0.161<br>136	0.166<br>141	0.064<br>55	0.340<br>287	0.325<br>275	0.324<br>274	0.324<br>273	0.323<br>273	0.078<br>67	0.307<br>258	0.326<br>275	0.327<br>274	0.312<br>263	0.310<br>261	0.310<br>261	0.315<br>265	0.319<br>268			0.316<br>266	0.334<br>283
**(6) *G. hamdii* sp. nov.**	0.085<br>69	0.097<br>79	0.100<br>81	0.149<br>119	0.297<br>224		0.138<br>126	0.133<br>122	0.354<br>302	0.354<br>302	0.341<br>293	0.186<br>168	0.137<br>129	0.149<br>139	0.150<br>140	0.145<br>135	0.343<br>294	0.155<br>143	0.173<br>157	0.181<br>164	0.128<br>117	0.129<br>118	0.127<br>116	0.171<br>155	0.169<br>153			0.134<br>123	0.026<br>25
**(7) *G. hanseni* sp. nov. _H1**	0.017<br>14	0.029<br>24	0.029<br>24	0.154<br>124	0.296<br>224	0.096<br>78		0.005<br>5	0.337<br>287	0.336<br>286	0.324<br>277	0.157<br>142	0.104<br>95	0.106<br>97	0.107<br>98	0.107<br>98	0.334<br>285	0.098<br>90	0.158<br>143	0.146<br>133	0.024<br>22	0.025<br>23	0.024<br>22	0.143<br>130	0.137<br>124			0.013<br>12	0.154<br>141
**(8) *G. hanseni* sp. nov. _H2**	0.013<br>11	0.026<br>21	0.026<br>21	0.156<br>125	0.296<br>224	0.091<br>74	0.006<br>5		0.337<br>287	0.335<br>285	0.326<br>278	0.153<br>138	0.101<br>92	0.103<br>94	0.104<br>95	0.104<br>95	0.335<br>286	0.095<br>87	0.153<br>139	0.142<br>129	0.021<br>19	0.022<br>20	0.021<br>19	0.139<br>126	0.132<br>120			0.010<br>9	0.149<br>137
**(9) *G. huyseae* sp. nov. _H1**	0.321<br>242	0.312<br>236	0.314<br>238	0.330<br>248	0.164<br>125	0.332<br>251	0.321<br>243	0.320<br>242		0.013<br>11	0.151<br>130	0.355<br>301	0.345<br>294	0.343<br>293	0.342<br>292	0.346<br>296	0.163<br>140	0.332<br>282	0.347<br>294	0.341<br>288	0.338<br>288	0.336<br>286	0.337<br>287	0.335<br>284	0.333<br>282			0.338<br>288	0.364<br>311
**(10) *G. huyseae* sp. nov. _H3**	0.321<br>242	0.312<br>236	0.314<br>238	0.332<br>249	0.168<br>128	0.334<br>252	0.323<br>244	0.320<br>242	0.010<br>8		0.154<br>132	0.356<br>302	0.344<br>293	0.342<br>292	0.341<br>291	0.345<br>295	0.162<br>139	0.332<br>282	0.347<br>294	0.341<br>288	0.336<br>286	0.335<br>285	0.336<br>286	0.335<br>284	0.333<br>282			0.336<br>286	0.363<br>310
**(11) *G. kuchtai* sp. nov.**	0.301<br>228	0.298<br>226	0.300<br>228	0.322<br>243	0.058<br>44	0.316<br>239	0.305<br>231	0.305<br>231	0.152<br>116	0.158<br>120		0.349<br>297	0.335<br>287	0.333<br>285	0.333<br>284	0.334<br>286	0.063<br>55	0.327<br>278	0.340<br>289	0.334<br>283	0.323<br>276	0.321<br>274	0.321<br>274	0.325<br>276	0.324<br>275			0.327<br>279	0.354<br>304
**(12) *G. lummei* sp. nov.**	0.131<br>106	0.130<br>105	0.132<br>107	0.171<br>137	0.313<br>236	0.154<br>124	0.135<br>109	0.130<br>105	0.341<br>257	0.341<br>257	0.322<br>243		0.152<br>137	0.152<br>138	0.153<br>138	0.157<br>142	0.343<br>292	0.144<br>130	0.067<br>62	0.117<br>107	0.153<br>138	0.155<br>140	0.155<br>140	0.110<br>100	0.106<br>97			0.155<br>140	0.203<br>184
**(13) *G. mendelensis* sp. nov.**	0.075<br>61	0.079<br>64	0.081<br>66	0.149<br>119	0.313<br>236	0.087<br>71	0.084<br>68	0.080<br>65	0.332<br>251	0.332<br>251	0.321<br>243	0.140<br>113		0.131<br>122	0.132<br>123	0.134<br>125	0.340<br>291	0.127<br>117	0.147<br>133	0.146<br>133	0.096<br>88	0.101<br>92	0.100<br>91	0.149<br>135	0.136<br>123			0.103<br>94	0.154<br>145
**(14) *G. prikrylovae* sp. nov.**	0.074<br>60	0.081<br>66	0.082<br>67	0.153<br>124	0.310<br>235	0.103<br>84	0.081<br>66	0.077<br>63	0.328<br>249	0.328<br>249	0.314<br>239	0.138<br>112	0.095<br>77		0.001<br>1	0.016<br>15	0.338<br>289	0.070<br>65	0.148<br>134	0.145<br>132	0.097<br>89	0.098<br>90	0.098<br>90	0.138<br>125	0.140<br>127			0.104<br>95	0.165<br>154
**(15) *G. prikrylovae* sp. nov._L1**	0.074<br>60	0.083<br>68	0.082<br>67	0.153<br>124	0.311<br>236	0.106<br>86	0.083<br>68	0.080<br>65	0.329<br>250	0.329<br>250	0.315<br>240	0.139<br>113	0.097<br>79	0.002<br>2		0.017<br>15	0.337<br>288	0.070<br>65	0.147<br>133	0.146<br>132	0.098<br>90	0.099<br>91	0.099<br>91	0.138<br>125	0.140<br>127			0.105<br>96	0.166<br>155
**(16) *G. scholzi* sp. nov.**	0.072<br>59	0.082<br>67	0.083<br>68	0.153<br>124	0.308<br>234	0.101<br>82	0.080<br>65	0.076<br>62	0.327<br>248	0.327<br>248	0.313<br>238	0.139<br>113	0.091<br>74	0.006<br>5	0.009<br>7		0.341<br>292	0.076<br>71	0.148<br>134	0.150<br>136	0.098<br>90	0.099<br>91	0.099<br>91	0.143<br>130	0.142<br>129			0.105<br>96	0.159<br>149
**(17) *G.* sp. ‘*C. neogaeus*’**	0.306<br>232	0.303<br>230	0.306<br>232	0.313<br>236	0.071<br>54	0.314<br>238	0.310<br>235	0.310<br>235	0.160<br>122	0.160<br>122	0.056<br>43	0.319<br>241	0.324<br>245	0.317<br>241	0.318<br>242	0.315<br>240		0.327<br>278	0.334<br>284	0.335<br>284	0.333<br>284	0.330<br>282	0.330<br>282	0.332<br>282	0.326<br>277			0.337<br>288	0.351<br>301
**(18) *G.* sp. ‘*H. nuchalis*’**	0.075<br>61	0.081<br>66	0.082<br>67	0.148<br>119	0.289<br>219	0.109<br>88	0.081<br>66	0.077<br>63	0.324<br>245	0.324<br>245	0.311<br>236	0.128<br>104	0.099<br>80	0.056<br>46	0.059<br>48	0.057<br>47	0.305<br>231		0.136<br>123	0.135<br>122	0.091<br>83	0.093<br>85	0.093<br>85	0.127<br>115	0.132<br>119			0.094<br>86	0.171<br>158
**(19) *G.* sp. 1 ‘*C. spadiceum*’**	0.128<br>104	0.123<br>100	0.126<br>102	0.173<br>139	0.304<br>229	0.141<br>114	0.135<br>110	0.131<br>106	0.337<br>254	0.337<br>254	0.320<br>242	0.056<br>46	0.127<br>103	0.135<br>110	0.136<br>111	0.131<br>107	0.317<br>240	0.127<br>103		0.106<br>97	0.149<br>135	0.152<br>138	0.151<br>137	0.091<br>83	0.090<br>82			0.155<br>141	0.192<br>174
**(20) *G.* sp. 2 ‘*C. spadiceum*’**	0.126<br>102	0.126<br>102	0.128<br>104	0.164<br>133	0.309<br>233	0.150<br>121	0.131<br>106	0.126<br>102	0.332<br>251	0.332<br>251	0.320<br>242	0.102<br>83	0.131<br>106	0.130<br>106	0.131<br>107	0.129<br>105	0.317<br>240	0.119<br>97	0.092<br>75		0.141<br>128	0.139<br>126	0.139<br>126	0.080<br>74	0.068<br>63			0.142<br>129	0.196<br>178
**(21) *G.* sp. 1 ‘*R. atratulus*’**	0.010<br>8	0.024<br>20	0.023<br>19	0.152<br>122	0.296<br>224	0.086<br>70	0.021<br>17	0.017<br>14	0.323<br>244	0.323<br>244	0.305<br>231	0.130<br>105	0.074<br>60	0.072<br>59	0.075<br>61	0.071<br>58	0.310<br>235	0.074<br>60	0.127<br>103	0.127<br>103		0.017<br>16	0.015<br>14	0.133<br>121	0.127<br>115			0.022<br>20	0.144<br>132
**(22) *G.* sp. 2 ‘*R. atratulus*’_L2**	0.011<br>9	0.021<br>17	0.021<br>17	0.149<br>120	0.291<br>220	0.087<br>71	0.021<br>17	0.017<br>14	0.320<br>242	0.320<br>242	0.301<br>228	0.133<br>108	0.079<br>64	0.075<br>61	0.077<br>63	0.074<br>60	0.305<br>231	0.075<br>61	0.129<br>105	0.123<br>100	0.015<br>12		0.003<br>3	0.138<br>125	0.132<br>120			0.023<br>21	0.144<br>132
**(23) *G.* sp. 2 ‘*R. atratulus*’_L3**	0.010<br>8	0.020<br>16	0.020<br>16	0.149<br>120	0.291<br>220	0.086<br>70	0.020<br>16	0.016<br>13	0.321<br>243	0.321<br>243	0.301<br>228	0.132<br>107	0.077<br>63	0.076<br>62	0.078<br>64	0.075<br>61	0.305<br>231	0.076<br>62	0.131<br>106	0.125<br>101	0.013<br>11	0.001<br>1		0.137<br>124	0.131<br>119			0.022<br>20	0.142<br>130
**(24) *G. spathulatus***	0.114<br>92	0.120<br>97	0.122<br>99	0.156<br>126	0.297<br>224	0.137<br>111	0.120<br>97	0.115<br>93	0.322<br>243	0.322<br>243	0.309<br>234	0.092<br>75	0.124<br>100	0.124<br>101	0.125<br>102	0.124<br>101	0.309<br>234	0.118<br>96	0.078<br>63	0.079<br>65	0.112<br>91	0.116<br>94	0.117<br>95		0.034<br>32			0.138<br>125	0.187<br>170
**(25) *G. stunkardi***	0.109<br>88	0.112<br>91	0.115<br>93	0.156<br>126	0.297<br>224	0.135<br>109	0.115<br>93	0.110<br>89	0.322<br>243	0.322<br>243	0.308<br>233	0.092<br>75	0.114<br>92	0.125<br>102	0.126<br>103	0.121<br>99	0.306<br>232	0.118<br>96	0.073<br>59	0.068<br>56	0.107<br>87	0.112<br>91	0.113<br>92	0.026<br>21				0.131<br>119	0.183<br>166
**(26) *Gyrodactylus* sp.[2]**	0.015<br>12	0.027<br>22	0.027<br>22	0.153<br>123	0.299<br>226	0.092<br>75	0.112<br>90	0.107<br>86	0.323<br>244	0.323<br>244	0.307<br>233	0.130<br>105	0.080<br>65	0.076<br>62	0.078<br>64	0.075<br>61	0.313<br>237	0.076<br>62	0.132<br>107	0.126<br>102	0.018<br>15	0.018<br>15	0.017<br>14	0.113<br>92	0.007<br>6				
**(27) *Gyrodactylus* sp.[3]**	0.105<br>84	0.109<br>88	0.111<br>89	0.156<br>125	0.303<br>227	0.135<br>108	0.010<br>8	0.006<br>5	0.326<br>244	0.326<br>244	0.312<br>234	0.092<br>74	0.118<br>95	0.124<br>100	0.124<br>100	0.120<br>97	0.313<br>235	0.119<br>96	0.072<br>58	0.072<br>59	0.104<br>84	0.109<br>88	0.111<br>89	0.020<br>16	0.109<br>88	0.106<br>85			
**(28) *G. variabilis***	0.015<br>12	0.029<br>24	0.027<br>22	0.156<br>125	0.302<br>228	0.095<br>77	0.012<br>10	0.009<br>7	0.325<br>246	0.325<br>246	0.310<br>235	0.132<br>107	0.082<br>67	0.078<br>64	0.078<br>64	0.077<br>63	0.315<br>239	0.079<br>64	0.134<br>109	0.128<br>104	0.021<br>17	0.021<br>17	0.020<br>16	0.116<br>94	0.111<br>90	0.107<br>86	0.002<br>2		0.150<br>138
**(29) *G. wardi***	0.086<br>70	0.097<br>79	0.100<br>81	0.154<br>123	0.296<br>223	0.007<br>6	0.097<br>79	0.092<br>75	0.333<br>251	0.332<br>250	0.317<br>240	0.155<br>125	0.086<br>70	0.105<br>85	0.107<br>87	0.103<br>84	0.316<br>239	0.110<br>89	0.145<br>117	0.147<br>119	0.088<br>71	0.088<br>71	0.086<br>70	0.139<br>112	0.134<br>108	0.134<br>107	0.094<br>76	0.096<br>78	

[1] *Gyrodactylus colemanensis* (JF836142) parasitizing *S. fontinalis* (Gilmore et al. 2012); [2] *Gyrodactylus* sp. (AY099508) parasitizing *R. osculus* (Boeger and Kritsky 2003); [3] *Gyrodactylus* sp. (KT149288) parasitizing *N. crysoleucas* (Leis et al. 2016).
