# Supplementary material for: Revealing the hidden diversity of Gyrodactylus communities (Monogenea, Gyrodactylidae) from Nearctic Catostomidae and Leuciscidae fish hosts (Teleostei, Cypriniformes), with descriptions of ten new species
Source: Parasite. 2023 Sep 27;30:40. doi: 10.1051/parasite/2023035 (PMC10537664; doi:10.1051/parasite/2023035)
Supplement: Supplementary file 2 — Table S2. Matrix of pairwise genetic distances (p-distance) based on the alignment of the 18S rDNA nucleotide sequences (435 bp long) (below diagonal) and number of variable nucleotides (above diagonal), including Gyrodactylus spp. investigated in this study (in bold) and their closest hits revealed by nBLAST search and listed in Table 2. H1 = L. chrysocephalus; H2 = S. atromaculatus; H3 = N. hudsonius (see Table 1 for fish sampling details). [file parasite-30-40-s2.pdf]

Table S2 Matrix of pairwise genetic distances (*p*–distance) based on the alignment of 18S rDNA nucleotide sequences (435 bp long) (below diagonal) and number of variable nucleotides (above diagonal), including *Gyrodactylus* species investigated in this study (**in bold**) and their closest hits revealed by nBLAST search and listed in Table 2. H1 = *L. chrysocephalus*; H2 = *S. atromaculatus*; H3 = *N. hudsonius* (see Table 1 for fish sampling details).

	**(1)**	(2)	**(3)**	(4)	**(5)**	**(6)**	**(7)**	**(8)**	**(9)**	**(10)**	**(11)**	**(12)**	**(13)**	**(14)**	**(15)**	**(16)**	**(17)**	**(18)**	**(19)**	**(20)**	**(21)**	**(22)**	**(23)**	(24)	**(25)**	(26)	**(27)**	(28)	**(29)**	**(30)**
**(1) *G. atratuli***		60	60	0	61	18	0	0	60	59	61	62	23	12	8	8	60	5	23	22	0	0	0	58	20	19	18	0	18	0
(2) *G. carassii*[1]	0,141		60	60	17	70	60	60	6	7	14	16	62	68	62	62	15	63	65	65	60	60	60	6	66	65	65	60	70	60
**(3) *G. colemanensis***	0,000	0,141		0	61	18	0	0	60	59	61	62	23	12	8	8	60	5	23	22	0	0	0	58	20	19	18	0	18	0
(4) *G. colemanensis*[2]	0,000	0,141	0,000		61	18	0	0	60	59	61	62	23	12	8	8	60	5	23	22	0	0	0	58	20	19	18	0	18	0
**(5) *G. ellae* n. sp.**	0,143	0,039	0,143	0,143		70	61	61	19	18	6	6	64	66	61	61	4	64	66	66	61	61	61	14	68	67	67	61	70	61
**(6) *G. hamdii* n. sp.**	0,043	0,166	0,043	0,043	0,166		18	18	69	68	71	72	33	26	23	23	69	21	32	32	18	18	18	67	32	31	29	18	1	18
**(7) *G. hanseni* n. sp._H1**	0,000	0,141	0,000	0,000	0,143	0,043		0	60	59	61	62	23	12	8	8	60	5	23	22	0	0	0	58	20	19	18	0	18	0
**(8) *G. hanseni* n. sp._H2**	0,000	0,141	0,000	0,000	0,143	0,043	0,000		60	59	61	62	23	12	8	8	60	5	23	22	0	0	0	58	20	19	18	0	18	0
**(9) *G. huyseae* n. sp._H1**	0,141	0,014	0,141	0,141	0,044	0,164	0,141	0,141		1	18	20	65	68	62	62	18	63	68	67	60	60	60	6	68	67	67	60	69	60
**(10) *G. huyseae* n. sp. _H3**	0,138	0,016	0,138	0,138	0,041	0,162	0,138	0,138	0,002		17	19	64	67	61	61	17	62	67	66	59	59	59	5	67	66	66	59	68	59
**(11) *G. kuchtai* n. sp.**	0,143	0,032	0,143	0,143	0,014	0,169	0,143	0,143	0,041	0,039		2	65	67	61	61	8	64	67	67	61	61	61	16	69	68	68	61	71	61
**(12) *G. laevisoides*[3]**	0,146	0,037	0,146	0,146	0,014	0,171	0,146	0,146	0,046	0,044	0,005		66	68	62	62	8	65	68	68	62	62	62	16	70	69	69	62	72	62
**(13) *G. lummei* n. sp.**	0,054	0,146	0,054	0,054	0,151	0,079	0,054	0,054	0,153	0,151	0,153	0,155		24	22	22	64	20	5	16	23	23	23	61	14	13	15	23	33	23
**(14) *G. mendeli* n. sp.**	0,028	0,160	0,028	0,028	0,155	0,062	0,028	0,028	0,160	0,157	0,157	0,160	0,056		14	14	67	13	23	25	12	12	12	66	22	21	19	12	26	12
**(15) *G. prikrylovae* n. sp.**	0,019	0,146	0,019	0,019	0,143	0,055	0,019	0,019	0,146	0,143	0,143	0,146	0,052	0,033		0	62	5	21	23	8	8	8	60	20	19	22	8	23	8
**(16) *G. scholzi* n. sp.**	0,019	0,146	0,019	0,019	0,143	0,055	0,019	0,019	0,146	0,143	0,143	0,146	0,052	0,033	0,000		62	5	21	23	8	8	8	60	20	19	22	8	23	8
**(17) *G.* sp. ‘*C. neogaeus*’**	0,141	0,035	0,141	0,141	0,009	0,164	0,141	0,141	0,041	0,039	0,018	0,018	0,151	0,157	0,146	0,146		63	67	65	60	60	60	12	67	66	66	60	69	60
**(18) *G.* sp. ‘*H. nuchalis’***	0,012	0,148	0,012	0,012	0,150	0,050	0,012	0,012	0,148	0,146	0,150	0,153	0,047	0,031	0,012	0,012	0,148		20	19	5	5	5	61	17	16	19	5	21	5
**(19) *G.* sp. 1 ‘*C. spadiceum’***	0,054	0,153	0,054	0,054	0,155	0,076	0,054	0,054	0,160	0,157	0,157	0,160	0,012	0,054	0,049	0,049	0,157	0,047		17	23	23	23	64	15	14	16	23	32	23
**(20) *G.* sp. 2 ‘*C. spadiceum’***	0,052	0,153	0,052	0,052	0,155	0,076	0,052	0,052	0,157	0,155	0,157	0,160	0,038	0,059	0,054	0,054	0,153	0,045	0,040		22	22	22	63	10	9	11	22	32	22
**(21) *G.* sp. 1 ‘*R. atratulus*’**	0,000	0,141	0,000	0,000	0,143	0,043	0,000	0,000	0,141	0,138	0,143	0,146	0,054	0,028	0,019	0,019	0,141	0,012	0,054	0,052		0	0	58	20	19	18	0	18	0
**(22) *G.* sp. 2 ‘*R. atratulus*’_1**	0,000	0,141	0,000	0,000	0,143	0,043	0,000	0,000	0,141	0,138	0,143	0,146	0,054	0,028	0,019	0,019	0,141	0,012	0,054	0,052	0,000		0	58	20	19	18	0	18	0
**(23) *G.* sp. 2 ‘*R. atratulus*’_2**	0,000	0,141	0,000	0,000	0,143	0,043	0,000	0,000	0,141	0,138	0,143	0,146	0,054	0,028	0,019	0,019	0,141	0,012	0,054	0,052	0,000	0,000		58	20	19	18	0	18	0
(24) *G. sedelnikowi*[4]	0,136	0,014	0,136	0,136	0,032	0,159	0,136	0,136	0,014	0,011	0,037	0,037	0,144	0,155	0,141	0,141	0,028	0,143	0,150	0,148	0,136	0,136	0,136		64	63	63	58	67	58
**(25) *G. spathulatus***	0,047	0,155	0,047	0,047	0,160	0,076	0,047	0,047	0,160	0,157	0,162	0,164	0,033	0,052	0,047	0,047	0,157	0,040	0,035	0,023	0,047	0,047	0,047	0,150		1	6	20	32	20
(26) *G. spathulatus*[5]	0,045	0,153	0,045	0,045	0,157	0,074	0,045	0,045	0,157	0,155	0,160	0,162	0,031	0,049	0,045	0,045	0,155	0,038	0,033	0,021	0,045	0,045	0,045	0,148	0,002		5	19	31	19
**(27) *G. stunkardi***	0,042	0,153	0,042	0,042	0,157	0,069	0,042	0,042	0,157	0,155	0,160	0,162	0,035	0,045	0,052	0,052	0,155	0,045	0,038	0,026	0,042	0,042	0,042	0,148	0,014	0,012		18	29	18
(28) *Gyrodactylus* sp.[6]	0,000	0,141	0,000	0,000	0,143	0,043	0,000	0,000	0,141	0,138	0,143	0,146	0,054	0,028	0,019	0,019	0,141	0,012	0,054	0,052	0,000	0,000	0,000	0,136	0,047	0,045	0,042		18	0
**(29) *G. variabilis***	0,043	0,166	0,043	0,043	0,166	0,002	0,043	0,043	0,164	0,162	0,169	0,171	0,079	0,062	0,055	0,055	0,164	0,050	0,076	0,076	0,043	0,043	0,043	0,159	0,076	0,074	0,069	0,043		18
**(30) *G. wardi***	0,000	0,141	0,000	0,000	0,143	0,043	0,000	0,000	0,141	0,138	0,143	0,146	0,054	0,028	0,019	0,019	0,141	0,012	0,054	0,052	0,000	0,000	0,000	0,136	0,047	0,045	0,042	0,000	0,043	

[1] *Gyrodactylus carassii* (AJ566377) parasitizing *A. alburnus* (Matějusová et al. 2003); [2] *Gyrodactylus colemanensis* (JF836090) parasitizing *S. fontinalis* (Gilmore et al. 2012);[3] *Gyrodactylus laevisoides* (KF263526) parasitizing *C. eos* (King et al. 2013); [4] *Gyrodactylus sedelnikovi* (AJ566378) parasitizing *B. barbatula* (Matějusová et al. 2003); [5] *Gyrodactylus spathulatus* (JF836098) parasitizing *C. commersonii* (Gilmore et al. 2012); [6] *Gyrodactylus* sp. (KT149284) parasitizing *N. crysoleucas* (Leis et al. 2016).
